# Advanced maternal age causes premature placental senescence and malformation via dysregulated α‐Klotho expression in trophoblasts

**DOI:** 10.1111/acel.13417

**Published:** 2021-06-09

**Authors:** Zhi Chen, Liling Xiong, Huili Jin, Jiaxiao Yu, Xin Li, Huijia Fu, Li Wen, Hongbo Qi, Chao Tong, Richard Saffery, Mark D. Kilby, Philip N. Baker

**Affiliations:** ^1^ Department of Obstetrics The First Affiliated Hospital of Chongqing Medical University Chongqing China; ^2^ State Key Laboratory of Maternal and Fetal Medicine of Chongqing Municipality Chongqing Medical University Chongqing China; ^3^ International Collaborative Laboratory of Reproduction and Development of Chinese Ministry of Education Chongqing Medical University Chongqing China; ^4^ Cancer, Disease and Developmental epigenetics, Murdoch Children's Research Institute Royal Children's Hospital Melbourne VIC Australia; ^5^ Centre for Women's and Newborn Health Institute of Metabolism and Systems Research University of Birmingham Birmingham UK; ^6^ College of Life Sciences University of Leicester Leicester UK

**Keywords:** advanced maternal age, placenta, senescence, trophoblast, α‐klotho

## Abstract

Advanced maternal age (AMA) pregnancy is associated with higher risks of adverse perinatal outcomes, which may result from premature senescence of the placenta. α‐Klotho is a well‐known antiaging protein; however, its expression and effect on the placenta in AMA pregnancies have not yet been fully elucidated. The expression patterns of α‐Klotho in mouse and human placentas from AMA pregnancies were determined by Western blotting and immunohistochemistry (IHC) staining. α‐Klotho expression in JAR cells was manipulated to investigate its role in trophoblastic senescence, and transwell assays were performed to assess trophoblast invasion. The downstream genes regulated by α‐Klotho in JAR cells were first screened by mRNA sequencing in α‐Klotho‐knockdown and control JAR cells and then validated. α‐Klotho‐deficient mice were generated by injecting *klotho*‐interfering adenovirus (Ad‐Klotho) via the tail vein on GD8.5. Ablation of α‐Klotho resulted in not only a senescent phenotype and loss of invasiveness in JAR cells but also a reduction in the transcription of cell adhesion molecule (CAM) genes. Overexpression of α‐Klotho significantly improved invasion but did not alter the expression of senescence biomarkers. α‐Klotho‐deficient mice exhibited placental malformation and, consequently, lower placental and fetal weights. In conclusion, AMA results in reduced α‐Klotho expression in placental trophoblasts, therefore leading to premature senescence and loss of invasion (possibly through the downregulation of CAMs), both of which ultimately result in placental malformation and adverse perinatal outcomes.

## Graphical abstract

Advanced maternal age is an independent risk factor for the loss of placental α‐Klotho expression beginning in the early phase of pregnancy. α‐Klotho deficiency in trophoblast not only induces senescence but also suppresses trophoblast invasion by downregulating the expression of cell adhesion molecules, both of which lead to maldevelopment of placenta, and ultimately result in higher risk of adverse perinatal outcomes

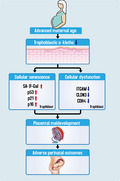

## 1 INTRODUCTION

Aging is defined as the deterioration of physiological functions that are critical for the survival and propagation of an organism as the organism grows older (Kuro‐o et al., 1997). Numerous diseases, such as arteriosclerosis, cancer, dementia, and chronic kidney disease, are reported to be associated with aging (Sopjani et al., 2015; Takahashi et al., 2000; Torbus‐Paluszczak et al., 2018). Although aging is a consequence of the interaction between environmental and genetic factors (Razzaque et al., 2006; Tsujikawa et al., 2003), few genes are deemed critical in the regulation of aging, including *Klotho*, which was originally identified because its mutation resulted in accelerated aging and shortened the lifespan in mice (Kuro‐o et al., 1997). The *Klotho* gene is located on chromosome 13q12 in both mice and humans (Torbus‐Paluszczak et al., 2018) and encodes the α‐Klotho protein. The absence of the α‐Klotho protein leads to the onset of several premature senescence phenotypes in mice, including infertility, arteriosclerosis, gait disturbances, cognitive decline, skin atrophy, and a shorter lifespan (Crasto et al., 2012; Kuro‐o et al., 1997; H. Liu et al., 2007; Semba et al., 2011; Semba et al., 2016; Shardell et al., 2016). However, the overexpression of α‐Klotho relieves these phenotypes and prolongs the lifespan of mice (Kurosu et al., 2005; Masuda et al., 2005). Thus, α‐Klotho has long been assumed to be a potent aging suppressor.

Emerging evidence has shown that α‐Klotho is widely expressed in human tissues and organs, including the kidney, parathyroid gland, small intestine, adipose tissue, and placenta (Imura et al., 2004). In humans, α‐Klotho expression levels are associated with age and begin to decline when a person reaches 40 years of age (Pedersen et al., 2013; Siahanidou et al., 2012; Yamazaki et al., 2010). Furthermore, α‐Klotho expression levels are decreased in patients with aging‐related diseases, such as cancer and hypertension (H. L. Wang et al., 2010; Y. Wang & Sun, 2009) and chronic kidney disease (Y. Wang & Sun, 2014). However, the antiaging mechanism of α‐Klotho in placentas and trophoblastic senescence has not yet been reported.

Advanced maternal age (AMA) is commonly defined as being 35 years or older at the time of delivery (Martinelli et al., 2018). In the last few decades, the incidence of AMA pregnancies has rapidly increased (Martin et al., 2019) due to socioeconomic factors and effective contraceptives (Cooke & Davidge, 2019), as well as improvements in assisted reproductive technologies (Blickstein, 2003). AMA pregnancies have higher risks of adverse perinatal outcomes, such as fetal loss, preterm birth, low birthweight, and preeclampsia (Abel et al., 2002; Jolly et al., 2000; Kortekaas et al., 2020; Sultana et al., 2018), all of which are believed to be due to premature placental senescence and consequent dysfunction. Senescence of the placenta is characterized by reduced telomerase activity (Biron‐Shental et al., 2010), increased DNA damage and DNA oxidation, and increased expression of senescent biomarkers [including tumor suppressor p53 and cyclin‐dependent kinase (CDK) inhibitors p16 and p21 (Londero et al., 2016)] and senescence‐associated secretory phenotype biomarkers IL‐6 and IL‐8 (Lu et al., 2017). Although numerous studies have reported that α‐Klotho is widely expressed in human placental tissue, the involvement of α‐Klotho in AMA‐related placental senescence has not yet been reported. In this study, we aimed to explore the role of α‐Klotho in regulating trophoblastic senescence in the context of AMA.

## 2 RESULTS

### 2.1 AMA is associated with placental senescence

**FIGURE 1:**
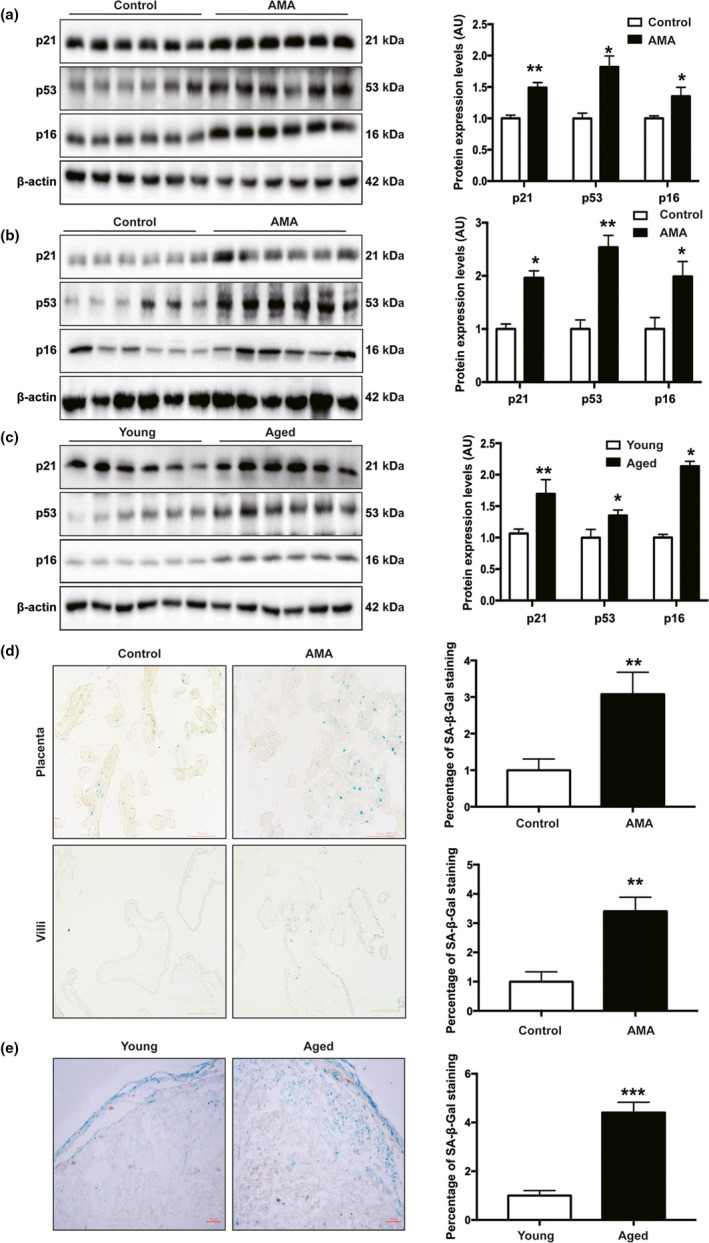
AMA placentas showed a senescence phenotype. (a) Western blotting of p21, p53, and p16 protein expression in human term placentas, *n* = 30 in the control and *n* = 37 in the AMA groups; (b) Western blotting of p21, p53 and p16 protein expression in human first trimester villi, *n* = 6 in each group; (c) Western blotting of p21, p53, and p16 protein expression in mouse placentas collected on GD18.5, *n* = 6; (d) representative images of SA‐β‐Gal staining of human term placenta sections. Quantification of the area of positive signal per sample (*n* = 3 patients per group, 3 random fields per patient). Scale bars, 100 μm; (e) representative images of SA‐β‐Gal staining of sections of mouse placentas collected on GD18.5. Quantification of the area of positive signal per mouse (*n* = 3 mice per group, 3 random fields per mouse). Scale bars, 100 μm. All data are presented as the mean ± SEM. *p < 0.05, **p < 0.01, ***p < 0.001. Mann–Whitney *U* test. NS, nonsignificant; AU, arbitrary unit. All experiments were performed in triplicate.

Human term placentas from AMA pregnancies showed significantly upregulated expression of multiple well‐known biomarkers of senescence, including p53, p21, and p16, compared with that in control placentas (Figure 1a). Interestingly, such elevations in p53, p21, and p16 expression in AMA placentas can be detected as early as the first trimester (Figure 1b). Similarly, the expression levels of p53, p21, and p16 in placentas from aged mice were also significantly higher than those in placentas from young mice (Figure 1c). In accordance with these findings, more positive senescence‐associated β‐galactosidase (SA‐β‐Gal) staining was observed in both term human placental tissues and first trimester villi of AMA pregnancies than in corresponding controls from young pregnancies (Figure 1d), while a similar manifestation was also observed in mouse placentas (Figure 1e). Taken together, these facts strongly indicate that AMA caused more severe senescence in the placenta, which started from the early stage of placentation.

### 2.2 AMA is associated with compromised α‐Klotho expression in the placenta

**FIGURE 2:**
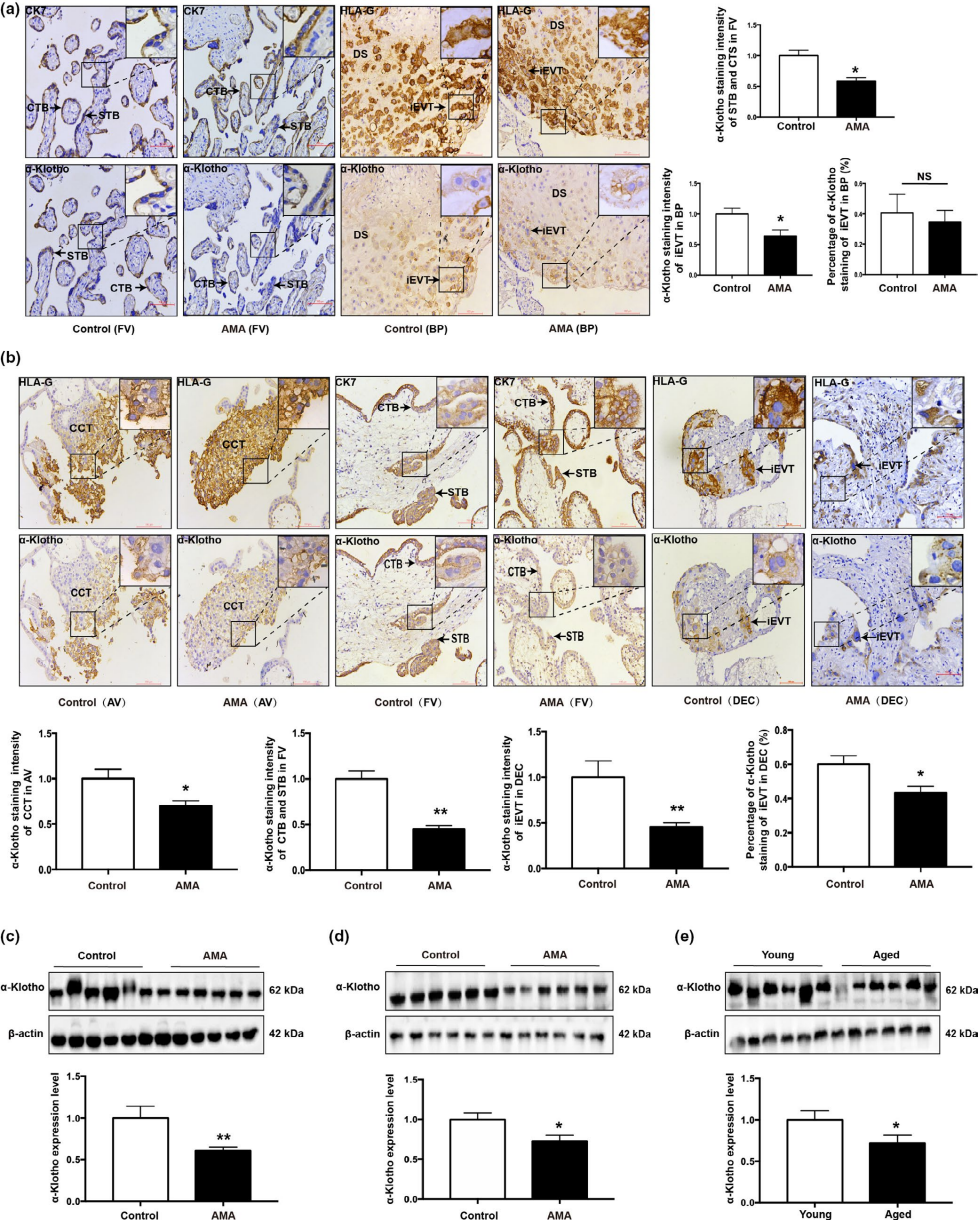
α‐Klotho expression pattern in human and mouse placentas. (a) IHC staining of α‐Klotho in human term placentas; Quantification of staining intensity per patient (*n* = 3 patients per group, 3 fields per patient); iEVTs and CTBs were identified by HLA‐G and CK7 staining, respectively; DS, decidual side; BP, basal plate; FV, floating villi; STB, syncytiotrophoblasts; iEVT, interstitial extravillous trophoblast; CTBs, cytotrophoblasts; CK7, cytokeratin 7; HLA‐G, human leukocyte antigen G. Scale bars: 100 μm; (b) IHC staining of α‐Klotho in human first trimester villi and decidua; quantification of staining intensity per patient (*n* = 3 patients per group, 3 fields per patient); AV, anchoring villi; iEVT, interstitial extravillous trophoblast; FV, floating villi; CCT, cell trophoblast; CK7, cytokeratin 7; HLA‐G, human leukocyte antigen G; DEC, decidua. Scale bars, 100 μm; (c) Western blotting of α‐Klotho protein expression in human term placentas, *n* = 30 in the control and *n* = 37 in the AMA groups; (d) Western blotting of α‐Klotho in human first trimester villi, *n* = 6; (e) Western blotting of α‐Klotho protein expression in mouse placentas collected on GD18.5, *n* = 6. All data are presented as the mean ± SEM. *p < 0.05, **p < 0.01, ***p < 0.001. Mann–Whitney *U* test. All experiments were performed in triplicate.

We then assessed the expression pattern of α‐Klotho in placental tissue. In humans, α‐Klotho is ubiquitously expressed in various types of placental trophoblast cells, including cytotrophoblasts (CTBs), cell column trophoblasts (CCT), syncytiotrophoblasts (STB), and interstitial extravillous trophoblast (iEVT) cells (Figure 2a, b & Figure S1,S2). Importantly, IHC staining also suggested that α‐Klotho expression was compromised in CTBs, STB in floating villi (FV), and iEVTs in BPs of term placentas and first‐trimester decidua collected from AMA pregnancies, which was confirmed by Western blotting (Figure 2c, d). Similarly, α‐Klotho was expressed in both the labyrinth zone (Lz) and junctional zone (Jz) areas in the mouse placenta (Figure S3). The reduction in placental α‐Klotho protein expression in human AMA pregnancies was confirmed in placentas collected from aged mice at GD18.5 compared with those of young controls (Figure 2e). Placental α‐Klotho expression remained stable throughout gestation in both humans and mice (Figure S4). Our findings suggest that AMA is associated with deficiency of α‐Klotho expression in placentas during placentation, which may be responsible for the senescence of the placenta.

### 2.3 α‐Klotho deficiency induces senescence in JAR cells and compromises its invasiveness

**FIGURE 3:**
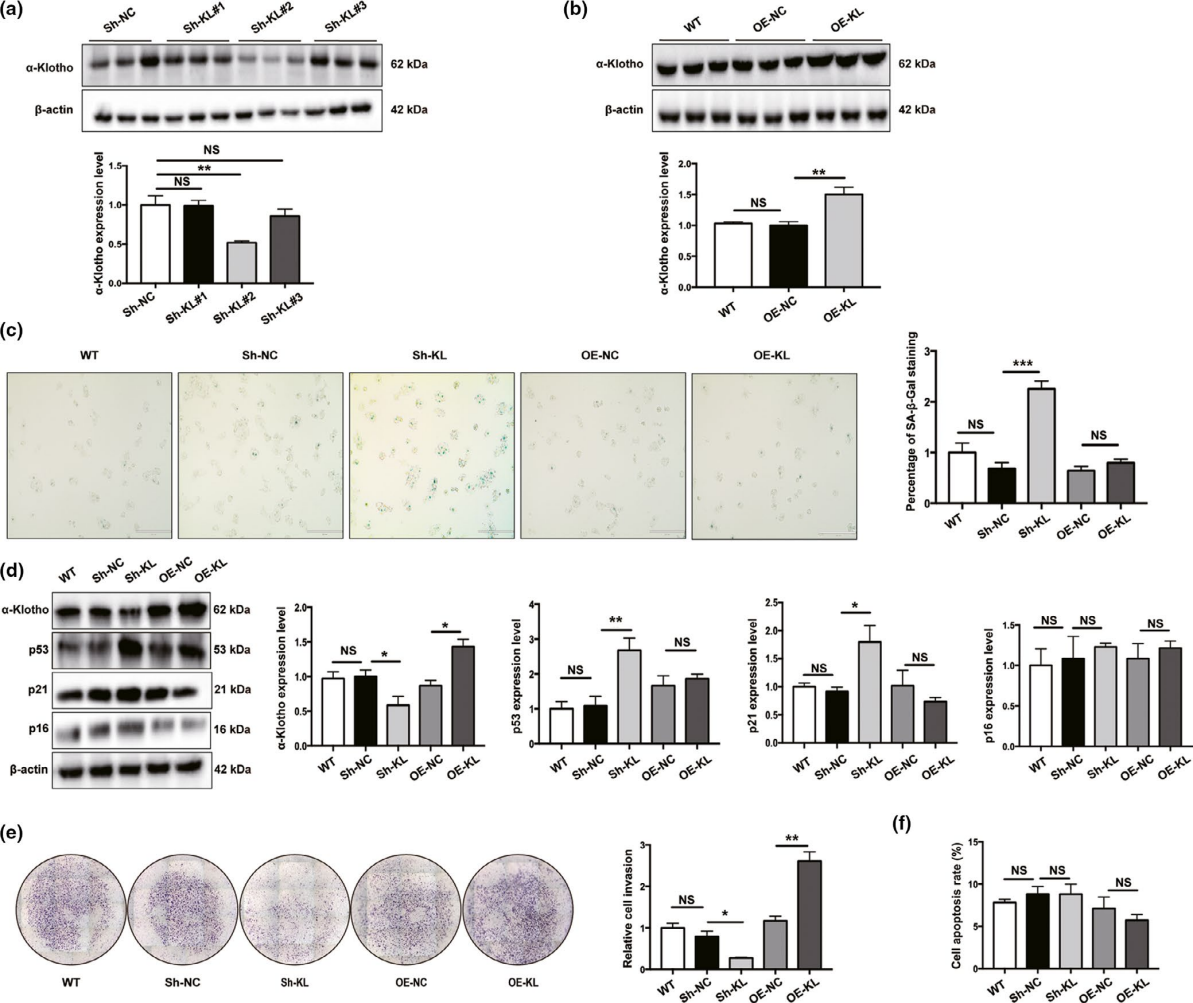
Loss of α‐Klotho‐induced premature senescence in JAR cells. (a) Western blotting of α‐Klotho in JAR cells. Sh‐NC, negative control cells transfected with scramble shRNA; Sh‐KL#1‐3, cells transfected with different shRNAs targeting α‐Klotho; (b) Western blotting of α‐Klotho protein expression in JAR cells. WT, wild‐type; OE‐NC, negative control; and OE‐KL, α‐Klotho overexpression; *n* = 3. (c) Representative SA‐β‐Gal staining in various JAR cells. Scale bars, 200 μm; (d) Western blotting of p53, p21, and p16 protein expression in Sh‐KL and OE‐KL JAR cells; (e) transwell invasion assay of JAR cells; (f) apoptosis was assessed using flow cytometry, *n* = 3. All data are presented as the mean ± SEM. *p < 0.05, **p < 0.01, ***p < 0.001, one‐way ANOVA. NS, nonsignificant. All experiments were performed in triplicate.

To investigate whether α‐Klotho is a determinant of senescence in placental trophoblasts, α‐Klotho‐knockdown (Sh‐KL) and overexpression (OE‐KL) JAR cells were generated, whereby α‐Klotho expression was repressed and elevated by nearly 50%, respectively (Figure 3a, 3b). Consistent with observations in human and mouse placentas, Sh‐KL JAR cells demonstrated higher levels of SA‐β‐gal than control JAR cells, while OE‐KL cells had SA‐β‐gal levels comparable to those of wild‐type and OE‐NC cells, both of which showed nearly undetectable expression (Figure 3c). Furthermore, the expression of p53 and p21 was increased in Sh‐KL cells, whereas p16 expression did not differ among the groups (Figure 3d). Invasive activity was diminished in Sh‐KL cells but significantly enhanced in OE‐KL cells (Figure 3e), but apoptosis rates of JAR cells were unaffected upon manipulation of α‐Klotho expression (Figure 3f). Furthermore, DNA synthesis among groups was not significantly different (Figure S5).

### 2.4 α‐Klotho‐deficient mice demonstrated a placental senescence phenotype and compromised placentation

**FIGURE 4:**
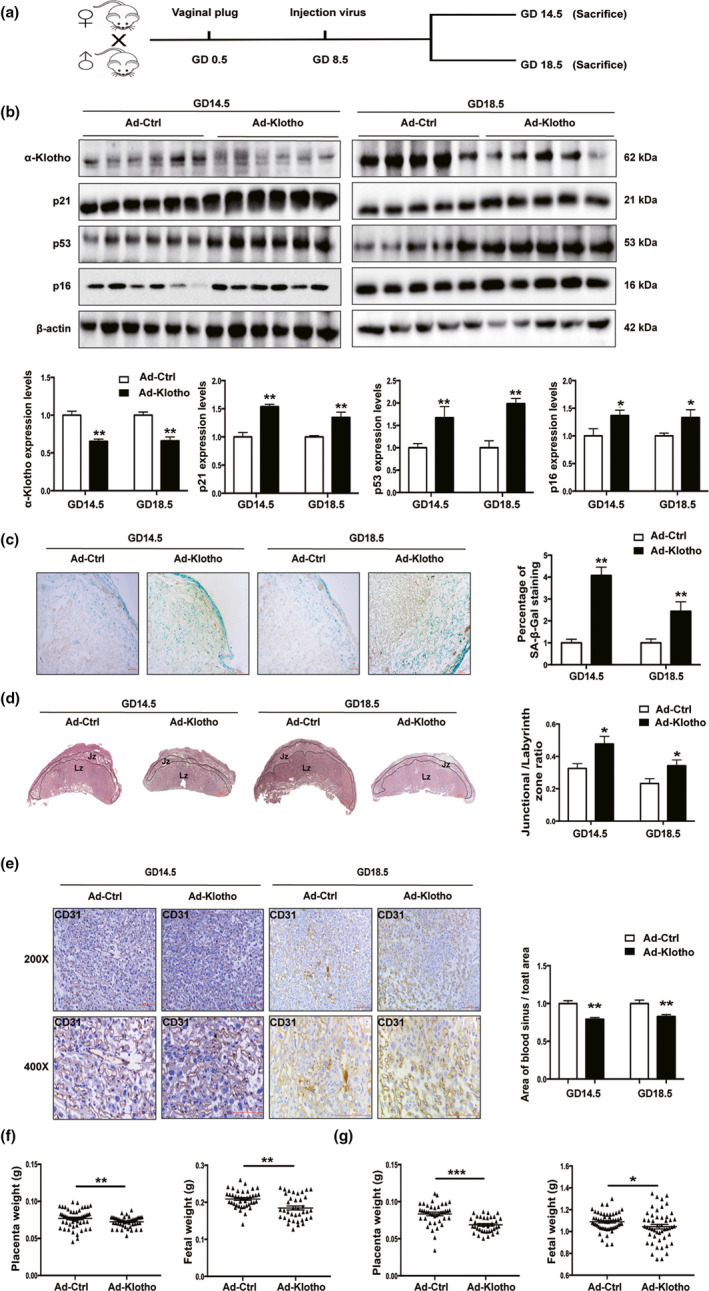
α‐Klotho deficiency leads to premature senescence and malformation of the mouse placenta. (a) Flow diagram of the experimental design; (b) Western blotting of α‐Klotho, p21, p53, and p16 protein expression in mouse placentas at GD14.5 (Ad‐Ctrl, *n* = 6; Ad‐Klotho, *n* = 6) and GD18.5 (Ad‐Ctrl, *n* = 5; Ad‐Klotho, *n* = 5); (c) representative images of SA‐β‐Gal staining of mouse placental sections at GD14.5 (Ad‐Ctrl, *n* = 6; Ad‐Klotho, *n* = 6) and GD18.5 (Ad‐Ctrl, *n* = 5; Ad‐Klotho, *n* = 5). Scale bars, 100 μm; (d) H&E staining of mouse placental sections at GD14.5 (Ad‐Ctrl, *n* = 6; Ad‐Klotho, *n* = 6) and GD18.5 (Ad‐Ctrl, *n* = 5; Ad‐Klotho, *n* = 5). The Lz and Jz areas and the Lz/Jz ratio were quantified at GD14.5 (Ad‐Ctrl, *n* = 6; Ad‐Klotho, *n* = 6) and GD18.5 (Ad‐Ctrl, *n* = 5; Ad‐Klotho, *n* = 5). Scale bars, 100 μm; (e) IHC staining of CD31 in the labyrinth of placentas collected from Ad‐Ctr or Ad‐Klotho mice on GD14.5 and GD18.5. The area of blood sinuses in the labyrinth was quantified using ImageJ 1.50i software, *n* = 3 per group, and 3 random fields per mouse were quantified. Scale bars: 100 μm; (f) placental weight and fetal weight on GD 14.5 (Ad‐Ctrl, *n* = 53 pups from 6 dams; Ad‐Klotho, *n* = 50 pups from 6 dams); (g) placental weight and fetal weight on GD 18.5 (Ad‐Ctrl, *n* = 40 pups from 5 dams; Ad‐Klotho, *n* = 38 pups from 5 dams). NS, nonsignificant. All data are presented as the mean ± SEM. *p < 0.05, **p < 0.01, ***p < 0.001. Mann‐Whitney *U* test. All experiments were performed in triplicate.

To verify our findings from the in vitro studies, α‐Klotho‐deficient mice were generated by injecting GFP‐tagged *klotho* interfering adenovirus (Ad‐ADPr‐klotho‐GFP, Ad‐Klotho) via the tail vein into pregnant females on GD8.5. The experimental design is depicted in Figure 4a. A significant reduction in placental α‐Klotho expression was observed on GD14.5 and GD18.5 (Figure 4b). As a result, the expression levels of p53, p21, and p16 were significantly elevated in the Ad‐Klotho placentas (Figure 4b). Accordingly, SA‐β‐gal staining was notably increased in the placentas of Ad‐Klotho mice compared with those of control mice at GD14.5 and GD18.5 (Figure 4c). Morphological assessments of the utero‐placental units revealed a marked reduction in the Lz area in the Ad‐Klotho placenta at GD14.5 and GD18.5 (Figure S6a), while the Jz area was not affected (Figure S6b). Consequently, the Jz/Lz area ratio was increased in α‐Klotho‐deficient placentas collected on either GD14.5 or GD18.5 (Figure 4d). More importantly, the area of blood sinuses in the Ad‐Klotho placenta at GD14.5 and GD18.5 was significantly compromised (Figure 4e), indicating maldevelopment of the placenta and compromised nutrient–oxygen exchange of the fetomaternal interface. Not surprisingly, α‐Klotho‐deficient mice exhibited a remarkable reduction in both placental and fetal weight throughout the later gestational stages (Figure 4f, 4g).

### 2.5 The cell adhesion molecule pathway plays a role in the regulation of senescence

**FIGURE 5:**
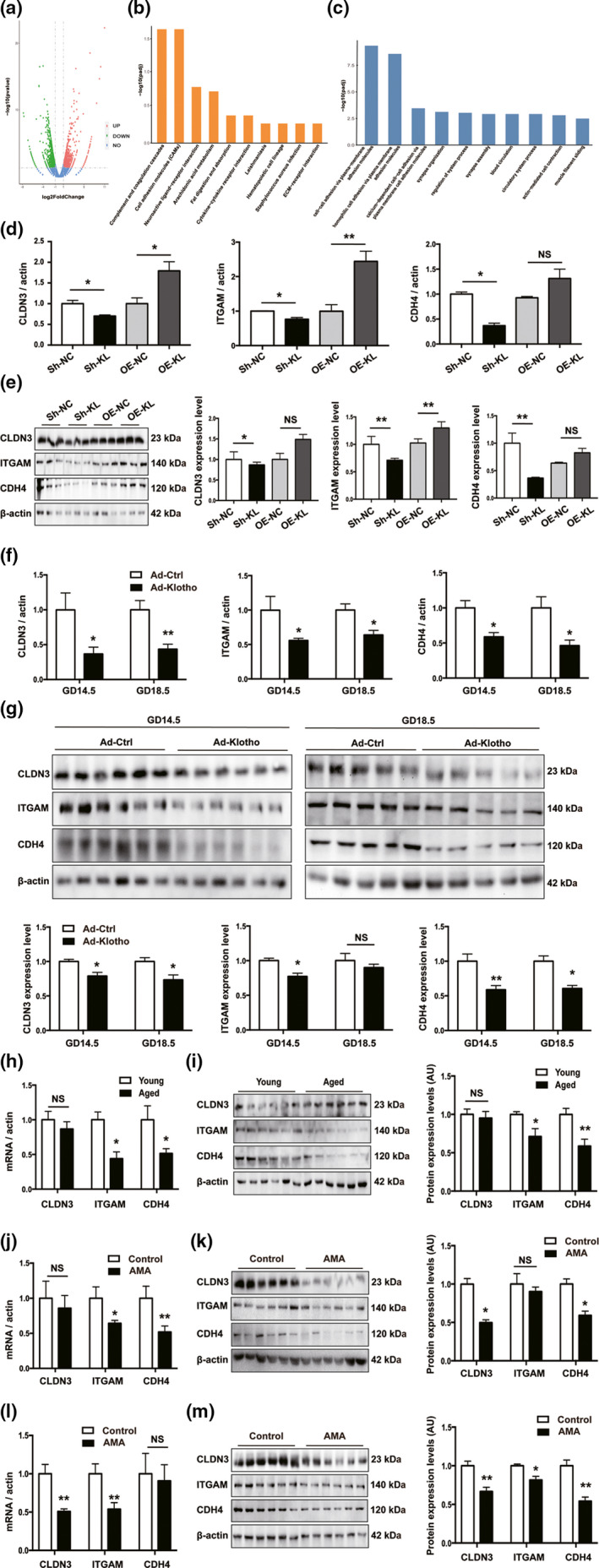
α‐Klotho regulates the genes of the CAM pathway in JAR cells. (a) Volcano plot of the significant differences in gene expression levels between Sh‐KL and Sh‐NC JAR cells; genes showing the greatest differences were analyzed by (b) KEGG and (c) GO analysis; (d‐e) mRNA levels and protein levels of CDH4, CLDN3, and ITGAM were examined separately by RT‐qPCR and Western blotting in various JAR cells, *n* = 3 per group; (f‐g) mouse placentas collected at different gestational ages: GD14.5 (Ad‐Ctrl, *n* = 6; Ad‐Klotho, *n* = 6) and GD18.5 (Ad‐Ctrl, *n* = 5; Ad‐Klotho, *n* = 5); (h‐i) placentas from young and aged mice on GD18.5, *n* = 6; (j‐k) human term placentas, protein levels (*n* = 6 per group), gene level, *n* = 7/8; and (l‐m) human first trimester villi, protein levels (*n* = 6 per group), gene level, *n* = 7/8. All data are presented as the mean ± SEM. *p < 0.05, **p < 0.01, ***p < 0.001. Mann–Whitney *U* test or one‐way ANOVA. NS, nonsignificant. All experiments were performed in triplicate.

To elucidate the mechanism underlying α‐Klotho deficiency‐induced senescence in placental trophoblasts, Sh‐KL and Sh‐NC JAR cells were subjected to mRNA sequencing. Compared with Sh‐NC cells, Sh‐KL cells showed significant alterations in the expression levels of 1876 mRNAs (1019 upregulated and 857 downregulated, Figure 5a). The disrupted genes were further enriched based on analysis of the Gene Ontology (GO) and Kyoto Encyclopedia of Genes and Genomes (KEGG) databases (Figure 5b, 5c; Figure S7). Notably, the cell adhesion molecule (CAM) pathway was downregulated via interference with α‐Klotho (Figure 5b), and the affected genes were involved in cell‐cell adhesion, as indicated by GO analysis (Figure 5c). The top 3 influenced CAM pathway genes were cadherin 4 (CDH4), claudin 3 (CLDN3), and integrin subunit alpha M (ITGAM), all of which were validated by RT‐qPCR and Western blot analysis. Our results revealed that the expression of these genes was significantly downregulated in Sh‐KL cells and accordingly upregulated in OE‐KL cells, except for that of CDH4, the expression of which was not altered upon overexpression of α‐Klotho (Figure 5d, 5e). In accordance with these data, α‐Klotho‐deficient mouse placentas demonstrated marked reductions in the gene expression of CDH4, CLDN3, and ITGAM (Figure 5f) on both GD14.5 and GD18.5, while similar changes in protein levels were further confirmed (Figure 5g). Moreover, GD18.5 placentas from aged mice were also associated with lower expression of ITGAM and GDH4 than gestational age‐matched placentas from young mice (Figure 5h, 5i). Consistently, the gene expression of CDH4 and ITGAM was decreased in term human placentas from the AMA group (Figure 5j), while the protein levels of CLDN3 and GDH4 were decreased in term human placentas from the AMA group (Figure 5k). Accordingly, mRNA resulting from CLDN3 and ITGAM expression was markedly reduced in the villi of AMA placentas during the first trimester (Figure 5l), while protein expression of CLDN3, CDH4, and ITGAM was markedly reduced in the villi of AMA placentas during the first trimester (Figure 5m). Nevertheless, the subcellular localization of CLDN3, CDH4, and ITGAM in human and mouse placentas did not vary (Figure S8, S9). Most importantly, interfering with CAM expression significantly impaired invasion in JAR cells (Figure S10).

## 3 DISCUSSION

Physiological aging of the placenta is an inevitable biological process (Sultana et al., 2018). However, aberrant or premature placental senescence disturbs the normal physiological function of the placenta, particularly the nutrient–oxygen exchange function, which could lead to adverse pregnancy outcomes (Cox & Redman, 2017; Sultana et al., 2018). α‐Klotho is a well‐known antiaging protein, and its expression declines with increasing age. However, the understanding of its role in placental senescence, especially in the context of AMA, is very limited. The present study revealed that α‐Klotho is expressed in various types of trophoblasts in the human placenta, which is consistent with previous results (Fan et al., 2016; Loichinger et al., 2016), implying that α‐Klotho expression possesses critical biological and pathological relevance in the placenta. Indeed, it has been reported that α‐Klotho expression levels in placentas from women diagnosed with preeclampsia or who delivered offspring that were small for gestational age (SGA) are significantly lower than those in placentas from women with healthy pregnancies (Fan et al., 2016; Iñiguez et al., 2018). α‐Klotho plays a role in the activation of the IGF‐I signaling pathway, which is vital for fetal growth (Iñiguez et al., 2018; Ohata et al., 2011) and may be associated with maintaining the balance of the fetomaternal calcium gradient (Ohata et al., 2011). Therefore, we speculated that these mechanisms may underlie the unfavorable birth outcomes associated with AMA.

The average maternal age of AMA subjects in this study was 40.86 years old, which is consistent with the reported age associated with a decline in α‐Klotho expression (Yamazaki et al., 2010). Studies have shown that α‐Klotho expression wanes in the skeletal muscle of aged male mice (Sahu et al., 2018) and aged human skin (Behera et al., 2017). These results indicated that the expression level of α‐Klotho may be associated with increased age. Previous findings from mouse muscle cells suggested that methylation of the *Klotho* gene promoter may be involved in the regulation of *Klotho* expression (Sahu et al., 2018); therefore, epigenetic modification could be the underlying mechanism of the downregulation of α‐Klotho expression in AMA trophoblasts.

Interestingly, our findings also indicated that gestational age had no effect on α‐Klotho expression, as placental α‐Klotho expression remained stable from the beginning to the end of gestation in both humans and mice. Moreover, in our animal experiments, paternal influence was excluded since all the female mice were bred with the same young male mouse. The results strongly suggested that AMA is an independent risk factor for the loss of placental α‐Klotho expression beginning in the early phase of pregnancy, implying that placental α‐Klotho expression is barely influenced by the life cycle of the placenta but is rather determined by maternal factors of the embryo. However, a previous study in another group showed that α‐Klotho expression in human placentas was downregulated as gestational age advanced (Iñiguez et al., 2018). The inconsistency between these findings may be due to the discrepancy in specimen collection. In our study, all human term placentas were collected from cesarean sections before parturition, whereas two‐thirds of the samples in Iñiguez's study were collected after natural delivery. Therefore, the effects of parturition and mechanistic stress from vaginal birth on placental α‐Klotho levels require further exploration.

To further investigate the effects of α‐Klotho deficiency on trophoblasts, we modulated α‐Klotho expression in JAR cells, which highly express α‐Klotho (Figure S11), and found that α‐Klotho deficiency promotes premature senescence, as reflected by the increased expression of p53 and p21. Interestingly, the expression of p16 in Sh‐KL JAR cells did not exhibit the upregulation observed in AMA placentas, possibly because p21 is downstream of p53 and is therefore expressed accordantly to mediate irreversible DNA damage and telomere shortening and additionally contribute to the stability and maintenance of cellular senescence. Our findings are consistent with a report that the downregulation of Klotho induces premature senescence of human primary fibroblast cells via a p53/p21‐dependent pathway (de Oliveira, 2006) without an increase in p16 expression. p16 mediates senescence through the retinoblastoma (Rb) pathway, inhibiting the action of cyclin‐dependent kinases and leading to G1 cell cycle arrest (Rayess et al., 2012). p16 is also a tumor suppressor (Romagosa et al., 2011) and can be overexpressed in tumor cells, such as human papilloma virus (HPV)‐related neoplasms (Milde‐Langosch et al., 2001). JAR cells are derived from choriocarcinoma (Hochberg et al., 1992; Zuo et al., 2018) and already maintain a relatively high basal expression level of p16; thus, p16 levels in JAR cells might be insensitive to α‐Klotho expression changes. Nevertheless, the use of only JAR cells is a limitation in our study. Future validation of our *in vitro* findings by using trophoblast stem cells is necessary.

In accordance with our results, α‐Klotho deficiency accelerated senescence in primary human fibroblast cells (MRC‐5) (de Oliveira, 2006). In contrast, supplementation with α‐Klotho expression relieves cellular senescence in human umbilical vein endothelial cells (HUVECs) and MRC‐5 cells, as shown by the decline in p53 and p21 expression (Ikushima et al., 2006). In this study, overexpression of α‐Klotho did not result in a significant reduction in either SA‐β‐gal staining or the expression of p53, p21, and p16, probably because of the high expression levels of α‐Klotho in JAR cells, which therefore maintains low expression levels of senescent biomarkers under basal conditions.

Nevertheless, the relationship between senescence and α‐Klotho expression in trophoblasts remains unclear. Therefore, we manipulated the senescence status of JAR cells and found that senescence was the result rather than the cause of α‐Klotho deficiency in trophoblasts (Figure S12). Moreover, the secretome of senescent trophoblast cells did not disturb α‐Klotho expression in adjacent cells (Figure S13).

Accumulating evidence suggests that the antiaging mechanism of α‐Klotho is possibly derived from but not limited to activating the fibroblast growth factor (FGF) 23 signaling pathway, which inhibits tissue atrophy by promoting cell proliferation and preventing cell death (Medici et al., 2008; Sugiura et al., 2005), drives the expression of the mitochondrial enzyme manganese superoxide dismutase (Mn‐SOD) by activating forkhead transcription factors (FoxO) and thus increases resistance to oxidative stress‐induced senescence (Mitobe et al., 2005; Yamamoto et al., 2005), mitigates cellular senescence in MRC‐5 cells and HUVECs by suppressing the p53/p21‐dependent pathway (Ikushima et al., 2006; de Oliveira, 2006), and inhibits inflammation mediated by retinoic acid‐inducible gene‐I (F. Liu et al., 2011).

In our study, we found that Sh‐KL JAR cells display a decline in invasion ability, and it has been established that placental trophoblast invasion is of particular importance in spiral artery remodeling, which is critical for placental function (Pijnenborg et al., 1980). Haram et al found that the adhesion molecule‐mediated invasion ability of trophoblasts is involved in the pathophysiology of preeclampsia (Haram et al., 2019). Therefore, we hypothesized that a decrease in invasion ability in placentas from AMA may be involved in pathophysiological placental development and activity. Intriguingly, despite the unchanged expression of p53, p21, p16, and SA‐β‐gal, JAR cell lines overexpressing α‐Klotho exhibited significantly improved invasive capability. This fact clearly suggests that α‐Klotho regulates JAR cells through a mechanism different from that of general biomarkers of senescence. Nonetheless, whether this regulatory mechanism of JAR cells is derived from their trophoblastic origin or tumor origin requires further investigation. Then, unbiased mRNA sequencing was performed, and the data demonstrated that CAMs, CDH4, CLDN3, and ITGAM are potential downstream target genes regulated by α‐Klotho in JAR cells. We then confirmed that CAM expression is required for the invasion of JAR cells, which is in accordance with previous reports noting that CDH4, CLDN3, and ITGAM are involved in the regulation of cell invasion. For example, Schumann et al reported that CLND3 was highly expressed in murine trophoblast giant cells and in human EVT cells and that it plays a role in the regulation of trophoblast invasion (Schumann et al., 2015; Zhao et al., 2020). Similarly, CDH4 deficiency impacts the invasion of human osteosarcoma cells (Tang et al., 2018), and epithelial ovarian cancer (EOC) invasion is mediated by ITGAM (Lyu et al., 2020). Moreover, the CAM pathway has been widely reported to be involved in regulating cell invasion (von Lersner et al., 2019; Thiery et al., 1988). Collectively, impaired α‐Klotho expression and consequent abnormal expression of adhesion molecules may be involved in the aberrant invasive activity of trophoblasts.

Although α‐Klotho−/− mice show a premature senescence phenotype and are widely used for the study of aging, their short lifespan (60.7 days) largely impedes their application in the study of AMA; therefore, the effects of α‐Klotho on the reproductive system, especially in the placenta, remain unclear. In this work, we generated an α‐Klotho‐deficient mouse model by injecting *klotho*‐interfering adenovirus (Ad‐Klotho) via the tail vein on GD8.5; this approach allows for the exclusion of the effects of α‐Klotho deficiency before placentation. In accordance with the results of cell experiments, the placentas of α‐Klotho‐deficient mice demonstrated a placental senescence phenotype. The mature placenta of the mouse consists of three parts: the labyrinth, spongiotrophoblast, and maternal decidua. The trophoblast and its associated fetal blood vessels form abundant branching to form a densely packed structure called the labyrinth (Watson & Cross, 2005). When the labyrinth develops, it is supported structurally by the spongiotrophoblast, which comprises a tight layer of nonsyncytial cells sandwiched between the labyrinth and the outer giant cells (Rossant & Cross, 2001). These structures are beneficial to the exchange of materials between the maternal and fetal environments. In the present study, α‐Klotho‐deficient mice exhibited an abnormal Jz/Lz area ratio indicative of placental malformation, which may be related to diminished transplacental nutrient transport and the consequent poor development of the fetus.

To our knowledge, this study is the first to report that AMA diminishes α‐Klotho in placental trophoblasts, therefore leading to premature senescence and loss of function, ultimately resulting in malformation of the placenta, which may contribute to placental deficiency and adverse perinatal outcomes. These findings demonstrated the importance of α‐Klotho in modulating placental senescence. To surmount senescence in the AMA placenta by targeting α‐Klotho, specific delivery of recombinant α‐Klotho into the placenta may be achievable via nanoparticles coated with placenta‐homing peptides (Tobita et al., 2017; Zhang et al., 2018).

## 4 MATERIALS AND METHODS

### 4.1 Patient and sample collection

Term placental tissue from women experiencing young pregnancies (20–25 years old, 37.14–41.14 weeks, *n* = 30) or AMA pregnancies (35–45 years old, 36.28–40.71 weeks, *n* = 37) who were admitted to the First Affiliated Hospital of Chongqing Medical University for cesarean sections was collected as previously described (J. E. Wagner et al., 1992). Patients with pregnancy complications, such as gestational diabetes mellitus, fetal growth restriction, spontaneous abortion, renal disease, or preeclampsia, were excluded. Young first‐trimester villi (20–25 years old, *n* = 20) and AMA first‐trimester villi (35–40 years old, *n* = 12) were collected from subjects who legally and voluntarily terminated their pregnancy between 6 and 10 weeks of gestational age. Patients with a history of spontaneous abortion or ectopic pregnancy were excluded.

A portion of the samples (100–200 g per Eppendorf tube) was washed with PBS, immediately frozen with liquid nitrogen, and stored at −80℃ until further use. A portion of the samples (30–50 mg per Eppendorf tube) was fixed overnight in 4% paraformaldehyde and then embedded in paraffin or optimal cutting temperature compound (OCT) for further use. The clinical characteristics of the patients are listed in Table S1‐S2. This study was approved by the Ethics Committee of the First Affiliated Hospital of Chongqing Medical University (No: 2020‐071) and in accordance with the principles outlined in the Declaration of Helsinki. Prior to their enrollment, the participants were fully briefed on the aim of this study. Written informed consent was obtained from all recruited subjects before sample collection.

### 4.2 Cell culture

The human choriocarcinoma cell line JAR was purchased from the Cell Bank of the Chinese Academy of Sciences (Shanghai, China). The JAR cells were cultured in Roswell Park Memorial Institute (RPMI) 1640 (Thermo Fisher Scientific, Waltham, MA, USA) medium supplemented with 10% fetal bovine serum (FBS) (Gibco, New York, USA) at 37℃ under 5% CO_2_ humidified air.

### 4.3 Lentivirus transfection

Lentiviruses carrying short hairpin RNA (shRNA) targeting human α‐Klotho were purchased from GenePharma (Shanghai, China). The sequences for the α‐Klotho knockdown virus are listed below: Sh‐KL#1 (5′‐GGATTGACCTTGAATTTAACC‐3′), Sh‐KL#2 (5′‐GCCAATTGGAATCTCCCAACC‐3′), Sh‐KL#3 (5′‐GCCAGGACAAGATGTTGTTGC‐3′), and Scrambled control shRNA (5′‐GTTCTCCGAACGTGTCACGT‐3′). Lentiviruses carrying the α‐Klotho overexpression vector were purchased from GeneChem (Shanghai, China). The sequences for the α‐Klotho overexpression virus are listed in Figure S14. A total of 1×10^5^ JAR cells were transfected with 2×10^6^ virus (multiplicity of infection = 20) for 48 hr in the presence of polybrene according to the instructions of the manufacturer. Two days after transfection, cells were treated with 2 µg/ml puromycin for 48 hr to select stably transfected cell clones. Flow cytometry was used to fluorescently screen cells that were successfully transfected with α‐Klotho overexpression virus. Knockdown and overexpression of α‐Klotho were verified by Western blot analysis.

### 4.4 siRNA silencing

Small interfering (si)–CLDN3 (Ma et al., 2019), si‐CDH4 (Xie et al., 2016), si‐ITGAM, and a negative control siRNA (si‐NC) were synthesized by GenePharma (Shanghai, China). Then, 50 nM siRNA was transfected into 60%‐70% of JAR cells in the presence of Lipofectamine 2000 (Thermo Fisher Scientific) in 6‐well plates according to the manufacturer's instructions. All cells were cultured for 48 hr after transfection before other treatments. Interference efficiency was verified by Western blot analysis. The siRNA sequences used in this study are listed in Table S3.

### 4.5 Immunohistochemistry

The villous and placental tissues were washed with PBS, fixed overnight with 4% paraformaldehyde at room temperature (RT), and sectioned into 4‐μm‐thick sections after they were dehydrated and embedded in paraffin. For immunohistochemistry (IHC) analysis, the tissue sections were deparaffinized and rehydrated in a graded alcohol series. Antigen retrieval was achieved by microwaving the sections in 10 mM citric sodium (pH 6.0) for 15 min. Then, the sections were treated with 3% H_2_O_2_ at RT for 15 min to block endogenous peroxidase activity. Next, the sections were incubated with rabbit mAb against α‐Klotho (1:100; AG27759; Proteintech, Rosemont, IL, USA), rabbit mAb against CLDN3 (1:100; AG9411; Proteintech), rabbit mAb against CDH4 (1:400; Proteintech), rabbit mAb against ITGAM (1:200; AG16327; Proteintech), mouse mAb against cytokeratin 7 (CK7) (1:100; EPR1619Y; Abcam, Cambridge, UK), mouse mAb against human leukocyte antigen G (HLA‐G) (1:100; MEM‐G/1; Proteintech), and rabbit mAb against CD31 (1:400; D8V9E; Cell Signaling Technology, USA) at 4℃ overnight, followed by treatment with a secondary antibody conjugated with horseradish peroxidase for 30 min at RT. The immunocomplexes were then visualized with diaminobenzidine. The images were captured under a light microscope (Leica Camera, Wetzlar, Germany). The positively stained intensity of each sample was quantified using ImageJ 1.50i software. Briefly, 3 random‐view fields of each sample in 3 independent experiments were quantified. The detailed quantitative procedures used in our study were derived from the published literature (Jensen, 2013). Furthermore, the area of blood sinuses in the labyrinth was quantified using ImageJ 1.50i software as previously reported (Varghese et al., 2014). Briefly, the positively stained area was divided by the total area. Three random‐view fields of each sample in 3 independent experiments were quantified.

### 4.6 RNA extraction and RT‐qPCR

Total RNA was extracted from JAR cells and placental tissues using TRIzol reagent (Invitrogen, USA). The RNA concentration was measured by ultraviolet spectroscopy (NanoDrop2000, Thermo, MA, USA). One microgram of total RNA was reverse transcribed to cDNA with a Prime Script RT reagent kit (Roche Life Science, Germany). The design and synthesis of primers were performed by TaKaRa (Dalian, China). SYBR Green dye (Roche) was used for real‐time PCR in an Applied Biosystems PCR cycler. The PCR primer sequences are shown in Table S4. The PCR cycling conditions were set as follows: predenaturation in a 96‐well plate at 95℃ for 10 min; 40 cycles of 95℃ for 10 s, 60℃ for 15 s, 72℃ for 15 s; and a final extension at 72℃ for 30 s. The mean threshold cycle (Ct) values were normalized to those of β‐actin, and the relative mRNA levels of ITGAM, CDH4, and CLDN3 were analyzed.

### 4.7 Western blotting

Total proteins were extracted using RIPA lysis buffer (Beyotime Biotechnology, Shanghai, China) supplemented with phenylmethylsulfonyl fluoride (PMSF) (Beyotime), and the protein concentration was determined with a BCA protein assay kit (Beyotime). The lysates were separated by SDS‐PAGE (Bio‐Rad, CA, USA) and transferred to PVDF membranes (Millipore Sigma, USA), which were blocked with 5% nonfat dry milk (Bio‐Rad) at RT for 1 hr before they were incubated with primary rabbit antibodies against p53 (1:1000; 1C12; CST), p21 (1:1000; EPR3993; Abcam), p16 (1:1000; EP1551Y; Abcam), β‐actin (1:1000; 2D4H5; Proteintech), CLDN3 (1:1000; AG9411; Abcam), CDH4 (1:500; Proteintech), ITGAM (1:1000; AG16327; Proteintech), and α‐Klotho (1:500; AG27759; Proteintech and 1:500; EPR6856; Abcam) overnight at 4℃. The membranes were washed and then incubated with horseradish peroxidase‐conjugated secondary rabbit antibodies (1:10,000; Proteintech) at RT for 1 hr. Protein bands were developed by enhanced chemiluminescent reagents (Millipore Sigma), and images were captured and analyzed with a Vilber Fusion image system (Fusion FX5 Spectra, France).

### 4.8 Apoptosis detection

Apoptosis was analyzed by flow cytometry using a BD FACSVantage SE Cell Sorter (BD Biosciences, San Jose, CA, USA). Briefly, JAR cells from different groups were collected and incubated with Annexin V–allophycocyanin (Thermo Fisher Scientific) and DAPI binding buffer (Thermo Fisher Scientific) for 20 min. The stained cells were then analyzed using a CytoFlex Flow Cytometer (BD Biosciences).

### 4.9 DNA synthesis assay

5‐Ethynyl‐2′‐deoxyuridine (EdU) assays were conducted using an EdU commercial Kit (Beyotime Biotechnology, China) according to the manufacturer's instructions. Briefly, cells at a density of 8000/well were seeded in 96‐well plates. Each well was supplemented with 100 μl of culture medium containing 50 μM EdU for 2 h. After being washed with PBS, cells were fixed with 4% formaldehyde for 30 min. After rewashing with 3% BSA containing 0.3% Triton X‐100 PBS, cells were incubated with a Click‐iTR EdU Kit for 30 min and then stained with Hoechst at RT for 30 min. Finally, images were captured using a fluorescence microscope (Evos Fl Color Imaging System), and the number of EdU‐positive cells was counted using ImageJ 1.50i software in 3 random‐view fields of each group in 3 independent experiments.

### 4.10 SA‐β‐Gal staining

SA‐β‐Gal staining was performed using a commercial kit (Senescence β‐Galactosidase Staining Kit, Beyotime) according to the manufacturer's instructions. In brief, the cells, villi, and placental tissues were fixed in fixative solution (formaldehyde‐glutaraldehyde mix) for 15 min at RT and stained overnight at 37℃ in a carbon dioxide‐free incubator. Staining was visualized by light microscopy, and images were captured (Leica camera). Blue signals were treated as positive signals, and the positively stained areas were quantified using ImageJ 1.50i software in 3 random‐view fields of each group in 3 independent experiments. Briefly, the area of positive signal per sample was quantified.

### 4.11 Matrigel invasion assay

The invasion assay was performed as previously reported by our laboratory (Yang et al., 2020). First, transwell inserts containing a polycarbonate membrane with an 8 μm pore size (Corning, New York, USA) were placed into 24‐well plates. The upper chamber was precoated with 60 μl of 1 mg/ml Matrigel matrix solution (BD Biosciences) before 8x10^4^ cells in 200 μl of serum‐free culture medium were added. The lower chamber contained 600 μl of culture medium supplemented with 10% FBS. After incubation for 24 hr, the inserts were fixed with 4% paraformaldehyde and stained with crystal violet. Images were captured using the Evos Fl Color Imaging System (Thermo Fisher Scientific). All data were analyzed using Image J 1.50i software.

### 4.12 Establishment of the AMA mouse model

Eight‐ to 12‐week‐old and 10‐ to 12‐month‐old virgin female C57BL/6J mice were purchased from Vital River Laboratory Animal Technology Company (Beijing, China) and designated as young and aged groups, respectively. All female mice were bred with one 8‐week‐old male C57BL/6J mouse. The day on which a vaginal plug was detected was considered GD0.5. All mice were kept in a temperature‐controlled room (23℃) with a 12:12 hr light–dark cycle. The mice were sacrificed on GD18.5 for further experiments. All animal procedures were approved by the Ethics Committee of the First Affiliated Hospital of Chongqing Medical University and conducted in accordance with the Guidelines of Chongqing Medical University.

### 4.13 Establishment of placental α‐Klotho‐deficient mice

Eight‐ to 12‐week‐old C57BL/6J virgin female mice were bred with age‐matched males. The day on which the vaginal plug was detected was considered GD0.5. The pregnant mice were randomly assigned into 2 groups: Adenovirus (Ad)‐Control (Ctrl) and Ad‐Klotho (*n* = 11 for both groups). EGFP‐tagged *klotho*‐interfering adenovirus was purchased from Biomedicine Biotech, Chongqing, China. On GD8.5, adenovirus was injected into pregnant mice via the tail vein (2 × 10^9^ PFU, 100 μl). The shRNA sequence used to target α‐Klotho was 5′‐GGTGGTTACCCTGTACCATTG‐3′. Six dams from each group were sacrificed on GD14.5, while the rest were sacrificed on GD18.5.

### 4.14 mRNA sequencing

Negative control and α‐Klotho‐knockdown JAR cells were collected for total RNA extraction (Invitrogen, USA). Total RNA was assessed by agarose gel electrophoresis for quality checks. Purity and integrity tests of RNA were conducted using a NanoPhotometer^®^ spectrophotometer (IMPLEN, USA) and an Agilent 2100 bioanalyzer (Agilent Technologies, US), respectively. A total of 1 μg of RNA from each sample served as input material for the sample preparations. Sequencing libraries were established using the NEBNext®UltraTM RNA Library Prep Kit for Illumina® (NEB, USA) according to the manufacturer's protocol. Differential expression analysis was performed using the DESeq2 R package (1.16.1). An adjusted P‐value of 0.05 and absolute fold‐change value of 2 served as the thresholds for significant differential expression. Differentially expressed genes were further analyzed by GO and KEGG database analyses.

### 4.15 Statistical analyses

The data are presented as the mean ± SEM. Statistical data were analyzed by the Mann–Whitney *U* test or one‐way ANOVA. A value of *p* < 0.05 was considered significant. The statistical analyses were performed using Prism7 software (GraphPad Software, La Jolla, CA, USA).

## CONFLICTS OF INTEREST

The authors have declared no conflicts of interest.

## AUTHOR CONTRIBUTIONS

CT and HQ conceived and designed the study; ZC, LX, HJ, JY, XL, HF, and LW performed the experiments and analyzed the data; MK, RS and PB interpreted the results; CT, LW and HQ provided funding resources; ZC wrote the draft; CT, MK, and PB edited the manuscript.

## Supporting information

Supplementary Material

## Data Availability

The data and materials described in the manuscript will be available upon reasonable request made to the corresponding authors, and delivery charges and agreement of usage may apply. The RNAseq data reported in this paper have been deposited in a public data depository under accession number HRA000693 and are publicly accessible at http://bigd.big.ac.cn/gsa‐human

## References

[acel13417-bib-0002] Abel, E. L. , Kruger, M. , & Burd, L. (2002). Effects of maternal and paternal age on Caucasian and Native American preterm births and birth weights. American Journal of Perinatology, 19(1), 49–54. 10.1055/s-2002-20173 11857096

[acel13417-bib-0003] Behera, R. , Kaur, A. , Webster, M. R. , Kim, S. , Ndoye, A. , Kugel, C. H. , Alicea, G. M. , Wang, J. , Ghosh, K. , Cheng, P. , Lisanti, S. , Marchbank, K. , Dang, V. , Levesque, M. , Dummer, R. , Xu, X. , Herlyn, M. , Aplin, A. E. , Roesch, A. , … Weeraratna, A. T. (2017). Inhibition of age‐related therapy resistance in melanoma by rosiglitazone‐mediated induction of klotho. Clinical Cancer Research, 23(12), 3181–3190. 10.1158/1078-0432.Ccr-17-0201 28232477 PMC5474161

[acel13417-bib-0004] Biron‐Shental, T. , Sukenik‐Halevy, R. , Sharon, Y. , Goldberg‐Bittman, L. , Kidron, D. , Fejgin, M. D. , & Amiel, A. (2010). Short telomeres may play a role in placental dysfunction in preeclampsia and intrauterine growth restriction. American Journal of Obstetrics and Gynecology, 202(4), 381.e381–387. 10.1016/j.ajog.2010.01.036 20350645

[acel13417-bib-0005] Blickstein, I. (2003). Motherhood at or beyond the edge of reproductive age. International Journal of Fertility and Women's Medicine, 48(1), 17–24. 12643516

[acel13417-bib-0006] Cooke, C. M. , & Davidge, S. T. (2019). Advanced maternal age and the impact on maternal and offspring cardiovascular health. American Journal of Physiology. Heart and Circulatory Physiology, 317(2), H387–h394. 10.1152/ajpheart.00045.2019 31199185

[acel13417-bib-0007] Cox, L. S. , & Redman, C. (2017). The role of cellular senescence in ageing of the placenta. Placenta, 52, 139–145. 10.1016/j.placenta.2017.01.116 28131318

[acel13417-bib-0008] Crasto, C. L. , Semba, R. D. , Sun, K. , Cappola, A. R. , Bandinelli, S. , & Ferrucci, L. (2012). Relationship of low‐circulating "anti‐aging" klotho hormone with disability in activities of daily living among older community‐dwelling adults. Rejuvenation Research, 15(3), 295–301. 10.1089/rej.2011.1268 22530731 PMC3388499

[acel13417-bib-0009] de Oliveira, R. M. (2006). Klotho RNAi induces premature senescence of human cells via a p53/p21 dependent pathway. FEBS Letters, 580(24), 5753–5758. 10.1016/j.febslet.2006.09.036 17014852

[acel13417-bib-0010] Fan, C. , Wang, Y. , Wang, J. , Lei, D. , Sun, Y. , Lei, S. , & Wang, S. (2016). Clinic significance of markedly decreased α‐klothoin women with preeclampsia. American Journal of Translational Research, 8(5), 1998–2010. 27347309 PMC4891414

[acel13417-bib-0012] Haram, K. , Mortensen, J. H. , Myking, O. , Magann, E. F. , & Morrison, J. C. (2019). The Role of Oxidative Stress, Adhesion Molecules and Antioxidants in Preeclampsia. Current Hypertension Reviews, 15(2), 105–112. 10.2174/1573402115666190119163942 30663572

[acel13417-bib-0014] Hochberg, A. , Rachmilewitz, J. , Eldar‐Geva, T. , Salant, T. , Schneider, T. , & de Groot, N. (1992). Differentiation of choriocarcinoma cell line (JAr). Cancer Research, 52(13), 3713–3717. 1617644

[acel13417-bib-0015] Ikushima, M. , Rakugi, H. , Ishikawa, K. , Maekawa, Y. , Yamamoto, K. , Ohta, J. , Chihara, Y. , Kida, I. , & Ogihara, T. (2006). Anti‐apoptotic and anti‐senescence effects of Klotho on vascular endothelial cells. Biochemical and Biophysical Research Communications, 339(3), 827–832. 10.1016/j.bbrc.2005.11.094 16325773

[acel13417-bib-0016] Imura, A. , Iwano, A. , Tohyama, O. , Tsuji, Y. , Nozaki, K. , Hashimoto, N. , Fujimori, T. , & Nabeshima, Y.‐I. (2004). Secreted Klotho protein in sera and CSF: implication for post‐translational cleavage in release of Klotho protein from cell membrane. FEBS Letters, 565(1–3), 143–147. 10.1016/j.febslet.2004.03.090 15135068

[acel13417-bib-0017] Iñiguez, G. , Gallardo, P. , Castro, J. J. , Gonzalez, R. , Garcia, M. , Kakarieka, E. , San Martin, S. , Johnson, M. C. , Mericq, V. , & Cassorla, F. (2018). Klotho Gene and Protein in Human Placentas According to Birth Weight and Gestational Age. Front Endocrinol (Lausanne), 9, 797. 10.3389/fendo.2018.00797 30697189 PMC6340928

[acel13417-bib-0018] Jensen, E. C. (2013). Quantitative analysis of histological staining and fluorescence using ImageJ. Anatomical Record (Hoboken), 296(3), 378–381. 10.1002/ar.22641 23382140

[acel13417-bib-0019] Jolly, M. , Sebire, N. , Harris, J. , Robinson, S. , & Regan, L. (2000). The risks associated with pregnancy in women aged 35 years or older. Human Reproduction, 15(11), 2433–2437. 10.1093/humrep/15.11.2433 11056148

[acel13417-bib-0020] Kortekaas, J. C. , Kazemier, B. M. , Keulen, J. K. J. , Bruinsma, A. , Mol, B. W. , Vandenbussche, F. , Van Dillen, J. , & De Miranda, E. (2020). Risk of adverse pregnancy outcomes of late‐ and postterm pregnancies in advanced maternal age: A national cohort study. Acta Obstetricia Et Gynecologica Scandinavica, 10.1111/aogs.13828 PMC7496606 32072610

[acel13417-bib-0021] Kuro‐o, M. , Matsumura, Y. , Aizawa, H. , Kawaguchi, H. , Suga, T. , Utsugi, T. , Ohyama, Y. , Kurabayashi, M. , Kaname, T. , Kume, E. , Iwasaki, H. , Iida, A. , Shiraki‐Iida, T. , Nishikawa, S. , Nagai, R. , & Nabeshima, Y.‐I. (1997). Mutation of the mouse klotho gene leads to a syndrome resembling ageing. Nature, 390(6655), 45–51. 10.1038/36285 9363890

[acel13417-bib-0022] Kurosu, H. , Yamamoto, M. , Clark, J. D. , Pastor, J. V. , Nandi, A. , Gurnani, P. , & Kuro‐o, M. (2005). Suppression of aging in mice by the hormone Klotho. Science, 309(5742), 1829–1833. 10.1126/science.1112766 16123266 PMC2536606

[acel13417-bib-0023] Liu, F. , Wu, S. , Ren, H. , & Gu, J. (2011). Klotho suppresses RIG‐I‐mediated senescence‐associated inflammation. Nature Cell Biology, 13(3), 254–262. 10.1038/ncb2167 21336305

[acel13417-bib-0024] Liu, H. , Fergusson, M. M. , Castilho, R. M. , Liu, J. , Cao, L. , Chen, J. , Malide, D. , Rovira, I. I. , Schimel, D. , Kuo, C. J. , Gutkind, J. S. , Hwang, P. M. , & Finkel, T. (2007). Augmented Wnt signaling in a mammalian model of accelerated aging. Science, 317(5839), 803–806. 10.1126/science.1143578 17690294

[acel13417-bib-0025] Loichinger, M. H. , Towner, D. , Thompson, K. S. , Ahn, H. J. , & Bryant‐Greenwood, G. D. (2016). Systemic and placental α‐klotho: Effects of preeclampsia in the last trimester of gestation. Placenta, 41, 53–61. 10.1016/j.placenta.2016.03.004 27208408 PMC5654625

[acel13417-bib-0026] Londero, A. P. , Orsaria, M. , Marzinotto, S. , Grassi, T. , Fruscalzo, A. , Calcagno, A. , Bertozzi, S. , Nardini, N. , Stella, E. , Lellé, R. J. , Driul, L. , Tell, G. , & Mariuzzi, L. (2016). Placental aging and oxidation damage in a tissue micro‐array model: an immunohistochemistry study. Histochemistry and Cell Biology, 146(2), 191–204. 10.1007/s00418-016-1435-6 27106773

[acel13417-bib-0027] Lu, L. , Kingdom, J. , Burton, G. J. , & Cindrova‐Davies, T. (2017). Placental stem villus arterial remodeling associated with reduced hydrogen sulfide synthesis contributes to human fetal growth restriction. American Journal of Pathology, 187(4), 908–920. 10.1016/j.ajpath.2016.12.002 PMC5397715 28157488

[acel13417-bib-0028] Lyu, T. , Jiang, Y. , Jia, N. , Che, X. , Li, Q. , Yu, Y. , & Feng, W. (2020). SMYD3 promotes implant metastasis of ovarian cancer via H3K4 trimethylation of integrin promoters. International Journal of Cancer, 146(6), 1553–1567. 10.1002/ijc.32673 31503345

[acel13417-bib-0029] Ma, L. , Yin, W. , Ma, H. , Elshoura, I. , & Wang, L. (2019). Targeting claudin‐3 suppresses stem cell‐like phenotype in nonsquamous non‐small‐cell lung carcinoma. Lung Cancer Manag, 8(1), Lmt04. 10.2217/lmt-2018-0010 31044015 PMC6488947

[acel13417-bib-0030] Martin, J. A. , Hamilton, B. E. , & Osterman, M. J. K. (2019). Births in the United States, 2018. NCHS Data Brief, 346, 1–8. 31442195

[acel13417-bib-0031] Martinelli, K. G. , Garcia, E. M. , Santos Neto, E. T. D. , & Gama, S. (2018). Advanced maternal age and its association with placenta praevia and placental abruption: a meta‐analysis. Cadernos de Saúde Pública, 34(2), e00206116. 10.1590/0102-311x00206116 29489954

[acel13417-bib-0032] Masuda, H. , Chikuda, H. , Suga, T. , Kawaguchi, H. , & Kuro‐o, M. (2005). Regulation of multiple ageing‐like phenotypes by inducible klotho gene expression in klotho mutant mice. Mechanisms of Ageing and Development, 126(12), 1274–1283. 10.1016/j.mad.2005.07.007 16144705

[acel13417-bib-0033] Medici, D. , Razzaque, M. S. , DeLuca, S. , Rector, T. L. , Hou, B. O. , Kang, K. , Goetz, R. , Mohammadi, M. , Kuro‐o, M. , Olsen, B. R. , & Lanske, B. (2008). FGF‐23‐Klotho signaling stimulates proliferation and prevents vitamin D‐induced apoptosis. Journal of Cell Biology, 182(3), 459–465. 10.1083/jcb.200803024 PMC2500132 18678710

[acel13417-bib-0034] Milde‐Langosch, K. , Bamberger, A. M. , Rieck, G. , Kelp, B. , & Löning, T. (2001). Overexpression of the p16 cell cycle inhibitor in breast cancer is associated with a more malignant phenotype. Breast Cancer Research and Treatment, 67(1), 61–70. 10.1023/a:1010623308275 11518467

[acel13417-bib-0035] Mitobe, M. , Yoshida, T. , Sugiura, H. , Shirota, S. , Tsuchiya, K. , & Nihei, H. (2005). Oxidative stress decreases klotho expression in a mouse kidney cell line. Nephron Exp Nephrol, 101(2), e67–74. 10.1159/000086500 15976510

[acel13417-bib-0036] Ohata, Y. , Arahori, H. , Namba, N. , Kitaoka, T. , Hirai, H. , Wada, K. , & Ozono, K. (2011). Circulating levels of soluble alpha‐Klotho are markedly elevated in human umbilical cord blood. Journal of Clinical Endocrinology and Metabolism, 96(6), E943–947. 10.1210/jc.2010-2357 21411554

[acel13417-bib-0037] Pedersen, L. , Pedersen, S. M. , Brasen, C. L. , & Rasmussen, L. M. (2013). Soluble serum Klotho levels in healthy subjects. Comparison of two different immunoassays. Clinical Biochemistry, 46(12), 1079–1083. 10.1016/j.clinbiochem.2013.05.046 23707222

[acel13417-bib-0038] Pijnenborg, R. , Dixon, G. , Robertson, W. B. , & Brosens, I. (1980). Trophoblastic invasion of human decidua from 8 to 18 weeks of pregnancy. Placenta, 1(1), 3–19. 10.1016/s0143-4004(80)80012-9 7443635

[acel13417-bib-0039] Rayess, H. , Wang, M. B. , & Srivatsan, E. S. (2012). Cellular senescence and tumor suppressor gene p16. International Journal of Cancer, 130(8), 1715–1725. 10.1002/ijc.27316 22025288 PMC3288293

[acel13417-bib-0040] Razzaque, M. S. , Sitara, D. , Taguchi, T. , St‐Arnaud, R. , & Lanske, B. (2006). Premature aging‐like phenotype in fibroblast growth factor 23 null mice is a vitamin D‐mediated process. The FASEB Journal, 20(6), 720–722. 10.1096/fj.05-5432fje 16436465 PMC2899884

[acel13417-bib-0041] Romagosa, C. , Simonetti, S. , López‐Vicente, L. , Mazo, A. , Lleonart, M. E. , Castellvi, J. , & Ramon y Cajal, S. (2011). p16(Ink4a) overexpression in cancer: a tumor suppressor gene associated with senescence and high‐grade tumors. Oncogene, 30(18), 2087–2097. 10.1038/onc.2010.614 21297668

[acel13417-bib-0042] Rossant, J. , & Cross, J. C. (2001). Placental development: lessons from mouse mutants. Nature Reviews Genetics, 2(7), 538–548. 10.1038/35080570 11433360

[acel13417-bib-0044] Sahu, A. , Mamiya, H. , Shinde, S. N. , Cheikhi, A. , Winter, L. L. , Vo, N. V. , Stolz, D. , Roginskaya, V. , Tang, W. Y. , St. Croix, C. , Sanders, L. H. , Franti, M. , Van Houten, B. , Rando, T. A. , Barchowsky, A. , & Ambrosio, F. (2018). Age‐related declines in α‐Klotho drive progenitor cell mitochondrial dysfunction and impaired muscle regeneration. Nature Communications, 9(1), 4859. 10.1038/s41467-018-07253-3 PMC6242898 30451844

[acel13417-bib-0045] Schumann, S. , Buck, V. U. , Classen‐Linke, I. , Wennemuth, G. , & Grümmer, R. (2015). Claudin‐3, claudin‐7, and claudin‐10 show different distribution patterns during decidualization and trophoblast invasion in mouse and human. Histochemistry and Cell Biology, 144(6), 571–585. 10.1007/s00418-015-1361-z 26340953

[acel13417-bib-0046] Semba, R. D. , Cappola, A. R. , Sun, K. , Bandinelli, S. , Dalal, M. , Crasto, C. , Guralnik, J. M. , & Ferrucci, L. (2011). Plasma klotho and cardiovascular disease in adults. Journal of the American Geriatrics Society, 59(9), 1596–1601. 10.1111/j.1532-5415.2011.03558.x 21883107 PMC3486641

[acel13417-bib-0047] Semba, R. D. , Ferrucci, L. , Sun, K. , Simonsick, E. , Turner, R. , Miljkovic, I. , Harris, T. , Schwartz, A. V. , Asao, K. , Kritchevsky, S. , & Newman, A. B. (2016). Low Plasma Klotho Concentrations and Decline of Knee Strength in Older Adults. Journals of Gerontology: Series A, Biological Sciences and Medical Sciences, 71(1), 103–108. 10.1093/gerona/glv077 PMC4706099 26359247

[acel13417-bib-0048] Shardell, M. , Semba, R. D. , Rosano, C. , Kalyani, R. R. , Bandinelli, S. , Chia, C. W. , & Ferrucci, L. (2016). Plasma Klotho and Cognitive Decline in Older Adults: Findings From the InCHIANTI Study. Journals of Gerontology: Series A, Biological Sciences and Medical Sciences, 71(5), 677–682. 10.1093/gerona/glv140 PMC5007737 26297657

[acel13417-bib-0049] Siahanidou, T. , Garatzioti, M. , Lazaropoulou, C. , Kourlaba, G. , Papassotiriou, I. , Kino, T. , Imura, A. , Nabeshima, Y.‐I. , & Chrousos, G. (2012). Plasma soluble α‐klotho protein levels in premature and term neonates: correlations with growth and metabolic parameters. European Journal of Endocrinology, 167(3), 433–440. 10.1530/eje-12-0476 22715479 PMC3638242

[acel13417-bib-0050] Sopjani, M. , Rinnerthaler, M. , Kruja, J. , & Dermaku‐Sopjani, M. (2015). Intracellular signaling of the aging suppressor protein Klotho. Current Molecular Medicine, 15(1), 27–37. 10.2174/1566524015666150114111258 25601466

[acel13417-bib-0051] Sugiura, H. , Yoshida, T. , Tsuchiya, K. , Mitobe, M. , Nishimura, S. , Shirota, S. , Akiba, T. , & Nihei, H. (2005). Klotho reduces apoptosis in experimental ischaemic acute renal failure. Nephrology, Dialysis, Transplantation, 20(12), 2636–2645. 10.1093/ndt/gfi165 16204278

[acel13417-bib-0052] Sultana, Z. , Maiti, K. , Dedman, L. , & Smith, R. (2018). Is there a role for placental senescence in the genesis of obstetric complications and fetal growth restriction? American Journal of Obstetrics and Gynecology, 218(2s), S762–s773. 10.1016/j.ajog.2017.11.567 29275823

[acel13417-bib-0053] Takahashi, Y. , Kuro, O. M. , & Ishikawa, F. (2000). Aging mechanisms. Proceedings of the National Academy of Sciences, 97(23), 12407–12408. 10.1073/pnas.210382097 PMC34062 11035783

[acel13417-bib-0054] Tang, Q. , Lu, J. , Zou, C. , Shao, Y. , Chen, Y. , Narala, S. , Fang, H. , Xu, H. , Wang, J. , Shen, J. , & Khokha, R. (2018). CDH4 is a novel determinant of osteosarcoma tumorigenesis and metastasis. Oncogene, 37(27), 3617–3630. 10.1038/s41388-018-0231-2 29610525

[acel13417-bib-0055] Thiery, J. P. , Boyer, B. , Tucker, G. , Gavrilovic, J. , & Valles, A. M. (1988). Adhesion mechanisms in embryogenesis and in cancer invasion and metastasis. Ciba Foundation Symposium, 141, 48–74. 10.1002/9780470513736.ch4 3075937

[acel13417-bib-0056] Tobita, T. , Kiyozumi, D. , & Ikawa, M. (2017). Placenta‐specific gene manipulation using lentiviral vector and its application. Placenta, 59(Suppl 1), S37–S43. 10.1016/j.placenta.2017.09.012 28988726

[acel13417-bib-0057] Torbus‐Paluszczak, M. , Bartman, W. , & Adamczyk‐Sowa, M. (2018). Klotho protein in neurodegenerative disorders. Neurological Sciences, 39(10), 1677–1682. 10.1007/s10072-018-3496-x 30062646 PMC6154120

[acel13417-bib-0058] Tsujikawa, H. , Kurotaki, Y. , Fujimori, T. , Fukuda, K. , & Nabeshima, Y. (2003). Klotho, a gene related to a syndrome resembling human premature aging, functions in a negative regulatory circuit of vitamin D endocrine system. Molecular Endocrinology, 17(12), 2393–2403. 10.1210/me.2003-0048 14528024

[acel13417-bib-0059] Varghese, F. , Bukhari, A. B. , Malhotra, R. , & De, A. (2014). IHC Profiler: an open source plugin for the quantitative evaluation and automated scoring of immunohistochemistry images of human tissue samples. PLoS One, 9(5), e96801. 10.1371/journal.pone.0096801 24802416 PMC4011881

[acel13417-bib-0060] von Lersner, A. , Droesen, L. , & Zijlstra, A. (2019). Modulation of cell adhesion and migration through regulation of the immunoglobulin superfamily member ALCAM/CD166. Clinical & Experimental Metastasis, 36(2), 87–95. 10.1007/s10585-019-09957-2 30778704 PMC6639050

[acel13417-bib-0061] Wagner, J. E. , Broxmeyer, H. E. , & Cooper, S. (1992). Umbilical cord and placental blood hematopoietic stem cells: collection, cryopreservation, and storage. Journal of Hematotherapy, 1(2), 167–173. 10.1089/scd.1.1992.1.167 1365024

[acel13417-bib-0063] Wang, H.‐L. , Xu, Q. , Wang, Z. , Zhang, Y.‐H. , Si, L.‐Y. , Li, X.‐J. , Yang, Q.‐H. , & Xiao, H. (2010). A potential regulatory single nucleotide polymorphism in the promoter of the Klotho gene may be associated with essential hypertension in the Chinese Han population. Clinica Chimica Acta, 411(5–6), 386–390. 10.1016/j.cca.2009.12.004 20005218

[acel13417-bib-0064] Wang, Y. , & Sun, Z. (2009). Klotho gene delivery prevents the progression of spontaneous hypertension and renal damage. Hypertension, 54(4), 810–817. 10.1161/hypertensionaha.109.134320 19635988 PMC2814175

[acel13417-bib-0065] Wang, Y. , & Sun, Z. (2014). Antiaging gene Klotho regulates endothelin‐1 levels and endothelin receptor subtype B expression in kidneys of spontaneously hypertensive rats. Journal of Hypertension, 32(8), 1629–1636. 10.1097/hjh.0000000000000233 24979306

[acel13417-bib-0066] Watson, E. D. , & Cross, J. C. (2005). Development of structures and transport functions in the mouse placenta. Physiology (Bethesda), 20, 180–193. 10.1152/physiol.00001.2005 15888575

[acel13417-bib-0067] Xie, J. , Feng, Y. , Lin, T. , Huang, X. Y. , Gan, R. H. , Zhao, Y. , & Lu, Y. G. (2016). CDH4 suppresses the progression of salivary adenoid cystic carcinoma via E‐cadherin co‐expression. Oncotarget, 7(50), 82961–82971. 10.18632/oncotarget.12821 27783992 PMC5347745

[acel13417-bib-0068] Yamamoto, M. , Clark, J. D. , Pastor, J. V. , Gurnani, P. , Nandi, A. , Kurosu, H. , & Kuro‐o, M. (2005). Regulation of oxidative stress by the anti‐aging hormone klotho. Journal of Biological Chemistry, 280(45), 38029–38034. 10.1074/jbc.M509039200 PMC2515369 16186101

[acel13417-bib-0069] Yamazaki, Y. , Imura, A. , Urakawa, I. , Shimada, T. , Murakami, J. , Aono, Y. , & Nabeshima, Y. (2010). Establishment of sandwich ELISA for soluble alpha‐Klotho measurement: Age‐dependent change of soluble alpha‐Klotho levels in healthy subjects. Biochemical and Biophysical Research Communications, 398(3), 513–518. 10.1016/j.bbrc.2010.06.110 20599764 PMC4130489

[acel13417-bib-0070] Yang, Y. , Xu, P. , Zhu, F. , Liao, J. , Wu, Y. , Hu, M. , & Baker, P. N. (2020). The potent antioxidant MITOQ protects against preeclampsia during late gestation but increases the risk of preeclampsia when administered in early pregnancy. Antioxidants & Redox Signaling, 10.1089/ars.2019.7891 32228063

[acel13417-bib-0071] Zhang, B. , Tan, L. , Yu, Y. , Wang, B. , Chen, Z. , Han, J. , & Fan, X. (2018). Placenta‐specific drug delivery by trophoblast‐targeted nanoparticles in mice. Theranostics, 8(10), 2765–2781. 10.7150/thno.22904 29774074 PMC5957008

[acel13417-bib-0072] Zhao, A. , Qi, Y. , & Liu, K. (2020). CLDN3 expression and function in pregnancy‐induced hypertension. Experimental Therapeutic Medicine, 20(4), 3798–3806. 10.3892/etm.2020.9084 32855729 PMC7444375

[acel13417-bib-0073] Zuo, Y. , Xu, Q. , Lu, Y. , Sun, D. , Wang, K. , Lei, Y. , & Li, Y. (2018). Dihydromyricetin induces apoptosis in a human choriocarcinoma cell line. Oncology Letters, 16(4), 4229–4234. 10.3892/ol.2018.9220 30214558 PMC6126223

