# Supplementary material for: Advanced maternal age causes premature placental senescence and malformation via dysregulated α‐Klotho expression in trophoblasts
Source: Aging Cell. 2021 Jun 9;20(7):e13417. doi: 10.1111/acel.13417 (PMC8282245; doi:10.1111/acel.13417)
Supplement: Supplementary file 1 — Supplementary Material [file ACEL-20-e13417-s001.docx]

**Supplementary Data**

**Table S1.** Clinical characteristics of subjects who provide placenta voluntarily.

| Characteristics | Control group  (n=30) | AMA group  (n=37) | *P*-value |
| --- | --- | --- | --- |
| Maternal age (years) | 23.06 (20-25) | 40.86 (35-45) | 0.000 |
| BMI (kg/m^2^) | 25.23 (23.13-27.60) | 25.56 (21.77-30.09) | 0.388 |
| Gestational weeks | 39.06 (37.14-41.14) | 38.73 (36.28-40.71) | 0.152 |
| Placental weight (g) | 639.39 (489.53-701.34) | 637.39 (501.56-689.14) | 0.612 |
| Gravidity | 1.36 (1-3) | 1.43 (1-2) | 0.588 |
| Neonatal birth weight (g) | 3325 (3120-3680) | 3281 (3019-3820) | 0.412 |

All data are presented as the median (range), and significant differences between groups were analyzed with Mann-Whitney U tests. P-value < 0.05 was considered a significant difference.

**Table S2.** Clinical characteristics of subjects who provide villi voluntarily.

| Characteristics | Control group  (n=20) | AMA group  (n=12) | *P*-value |
| --- | --- | --- | --- |
| Maternal age (years) | 22.09 (20-25) | 37.08 (35-40) | 0.000 |
| BMI (kg/m^2^) | 24.88 (20.98-29.79) ± | 25.35 (24.16-29.18) | 0.508 |
| Gestational weeks | 7.26 (6-10.43) | 7.04 (6-10.29) | 0.249 |
| Gravidity | 1.15 (1-2) | 1.33 (1-2) | 0.232 |

All data are presented as the median (range), and significant differences between groups were analyzed with Mann-Whitney U tests. P-value < 0.05 was considered a significant difference.

**Table S3.** The si-RNA sequences used in this study.

|  | Sequences (5＇→3＇) |
| --- | --- |
| si-NC | 5'-UUCUCCGAACGUGUCACGUTT-3' |
| si-ITGAM | 5′-GAAUUCCGGAUUCACUUUATT-3′ |
| si-CDH4  si-CLDN3 | 5′-CAGUCGACUACGAGCUCAATT-3′  5′-GCUACGACCGCAAGGACUA-3′ |

**Table S4.** Sequences of primers used in this study.

| Primers (mouse) | Sequences (5＇→3＇) |
| --- | --- |
| ITGAM | Forward TTCTTGCCTGTGACCAATGCC |
|  | Reverse TTGCCCAGAGAAGGACCCAT |
| CDH4 | Forward GCCTCTTACCATCTCCGAGCC  Reverse CCTCATCCACAGAGCCGTTG |
| CLDN3  β-actin | Forward CTACCAGCAGTCGATGAACCC  Reverse GTCCTCTTCCAGCCTAGCAAG  Forward TGTGACGTTGACATCCGTAAAG  Reverse TCAGTAACAGTCCGCCTAGAA |

| Primers (human) | Sequences (5＇→3＇) |
| --- | --- |
| ITGAM | Forward AGGCAATCATGGAGTTCAATCCC |
|  | Reverse ATCTGTCCTTCTCTTAGCCGAT |
| CDH4 | Forward TTCAAACCCCAGGACACTCTCG  Reverse CACGCCCAACCGTAACTGC |
| CLDN3  β-actin | Forward CTGCTCTGCTGCTCGTGTCCC  Reverse GGTCTCCCTGCGTCTGTCCC  Forward TGGCACCCAGCACAATGAA  Reverse CTAAGTCATAGTCCGCCTAGAAGCA |


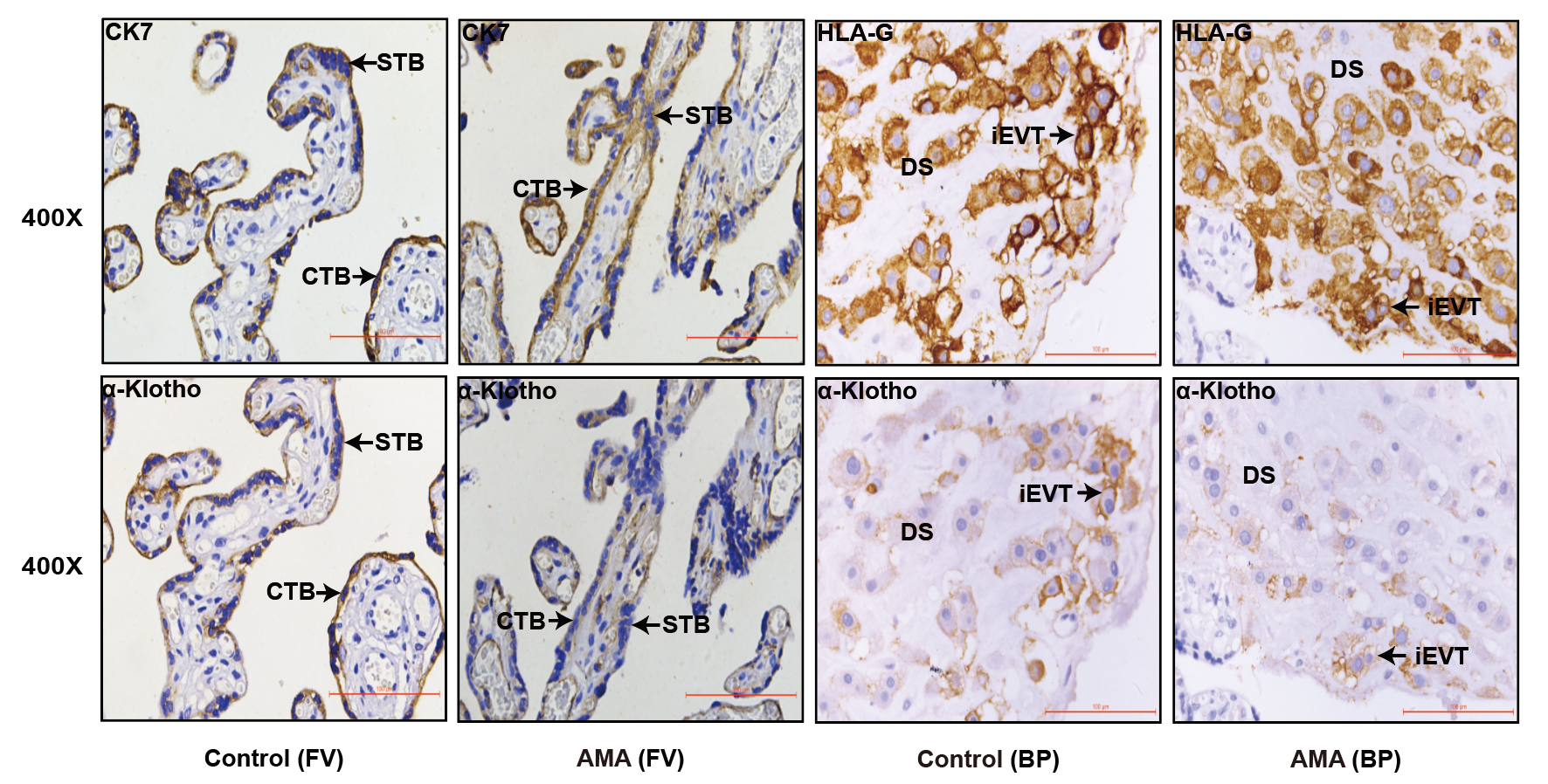


**Figure.S1 IHC staining of α-Klotho in human term placentas.**

iEVTs and CTBs were identified by HLA-G and CK7 staining, respectively; DS, decidual side; BP, basal plate; FV, floating villi; iEVT, interstitial extravillous trophoblast; STBs, syncytiotrophoblasts; CTBs, cytotrophoblast; CK7, cytokeratin 7; HLA-G, human leukocyte antigen G. Scale bars: 100μm. 400X magnification images were depicted.


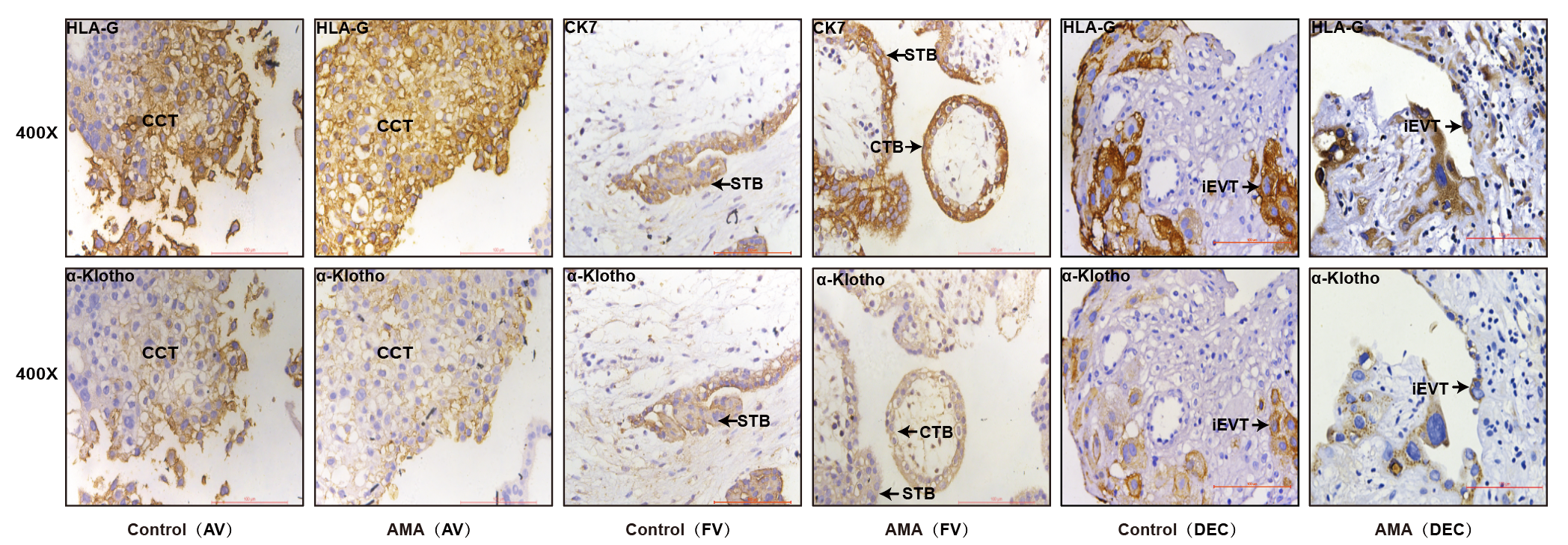


**Figure.S2** **IHC staining of α-Klotho in human first trimester villi and decidua.**

iEVTs and CTBs were identified by HLA-G and CK7 staining, respectively; DS, decidual side; BP, basal plate; FV, floating villi; iEVT, interstitial extravillous trophoblast; STBs, syncytiotrophoblasts; CTBs, cytotrophoblast; CK7, cytokeratin 7; HLA-G, human leukocyte antigen G. Scale bars: 100μm. 400X magnifications images were depicted.


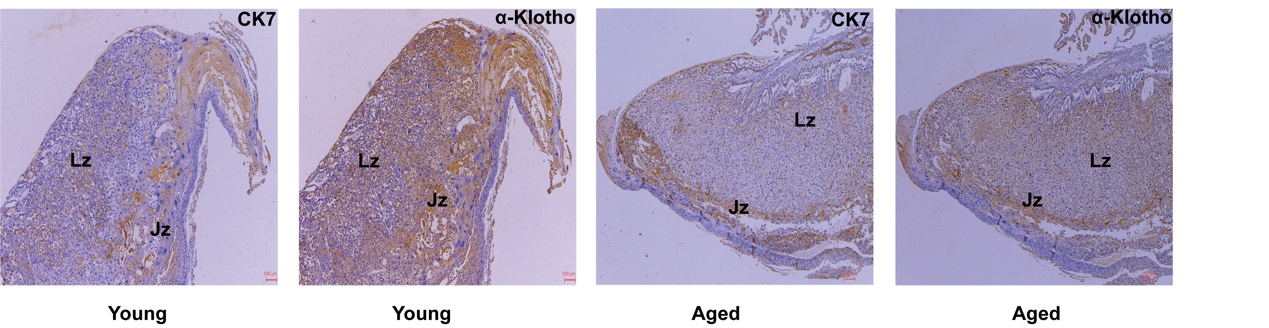


**Figure.S3** **α-Klotho expression in mouse term placentas.**

IHC staining of α-Klotho in placentas collected from young and aged mice on GD18.5 on serial sections. Lz and Jz were stained by CK7; Lz, labyrinth zone area, Jz, junctional zone area. scale bars, 100μm.

**a**

**
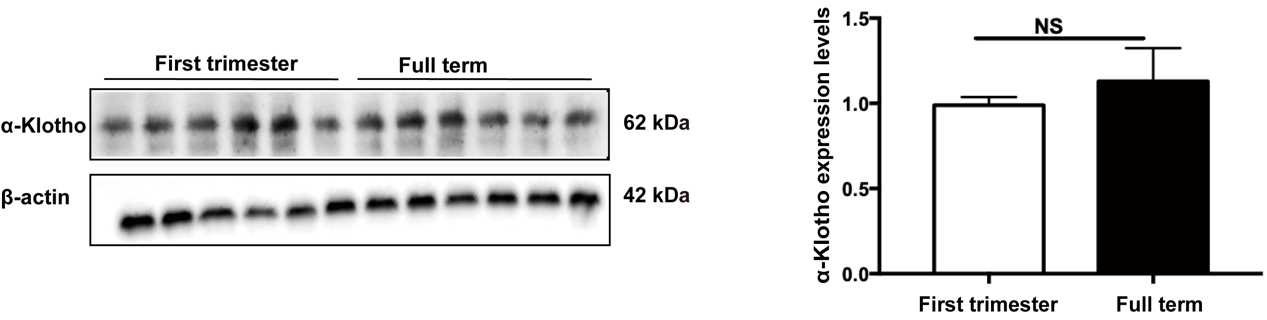
**

**b**

**
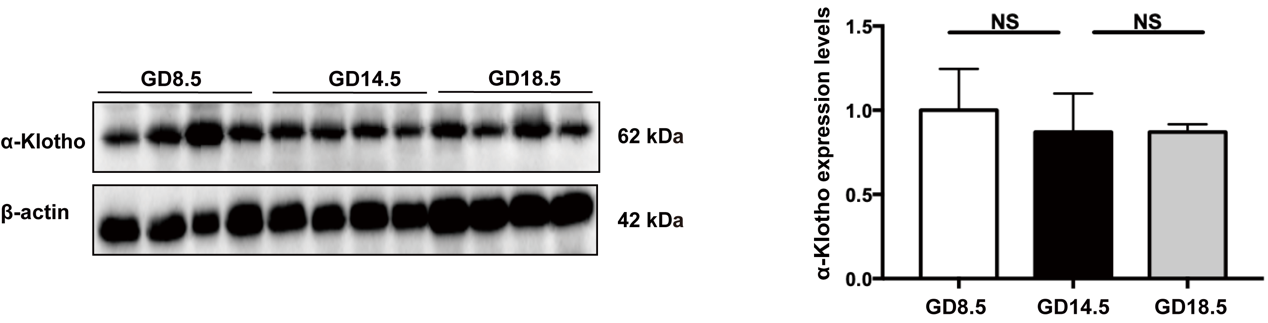
**

**Figure.S4** **The impact of gestational age on placental** **α-Klotho expression pattern.**

(a) Western blotting of α-Klotho in the first trimester human villi and full-term placentas of normal pregnancies, n=6. Data are means ± SEM, NS, nonsignificant. (b) Western blotting of α-Klotho in mouse term placentas on GD8.5, GD14.5 and GD18.5, n=3. All data are presented as the means ± SEM. *P, < 0.05, **P, <0.01, ***P, <0.001. Mann-Whitney U test. All experiments are performed in triplicate.


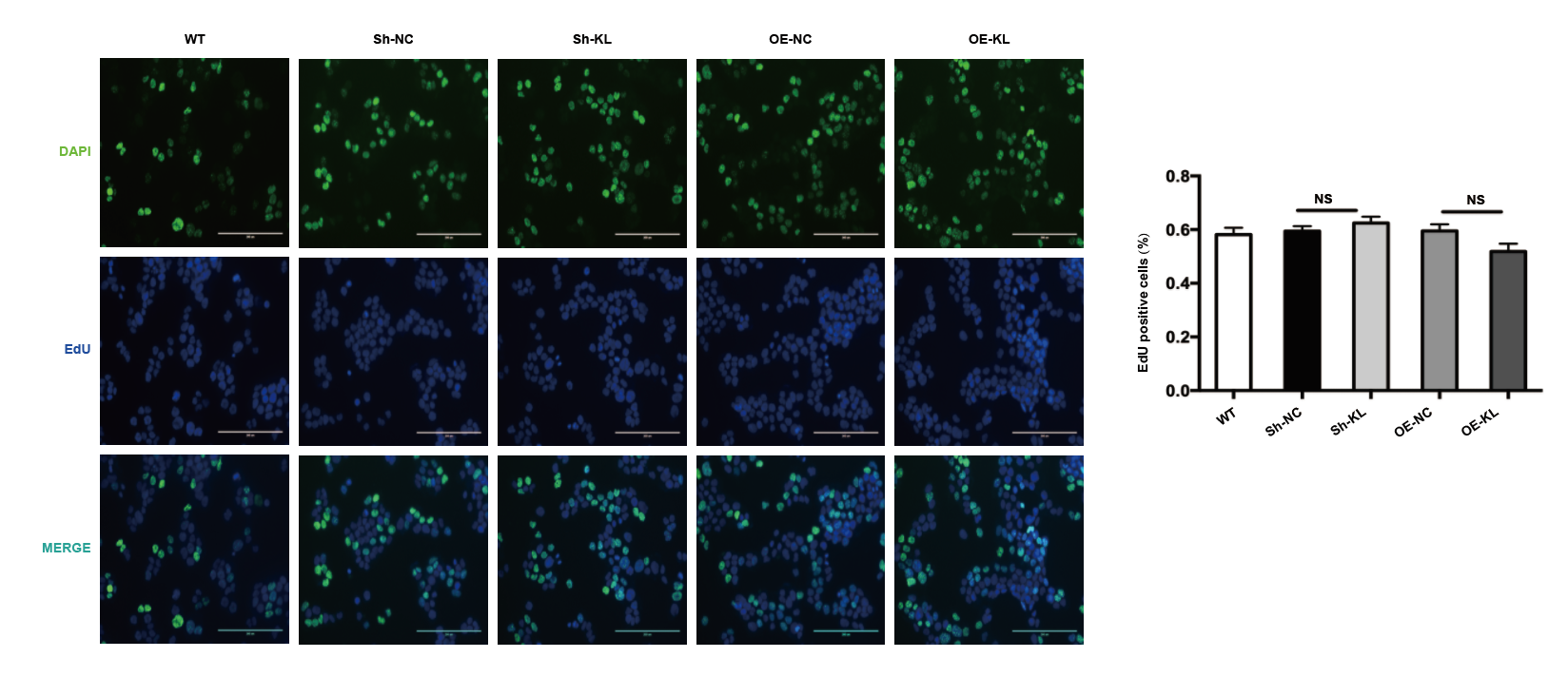


**Figure S5 EDU staining in JAR cells**

EDU staining in various JAR cells. WT, wild-type; Sh-NC, negative control cells transfected with scramble shRNA; OE-NC, negative control; Sh-KL, cells transfected with shRNA targeting α-Klotho and OE-KL, α-Klotho overexpression; n=3 in per group. All data are presented as the means ± SEM. One-way ANOVA. NS, nonsignificant. All experiments were performed in triplicate.

**a b**


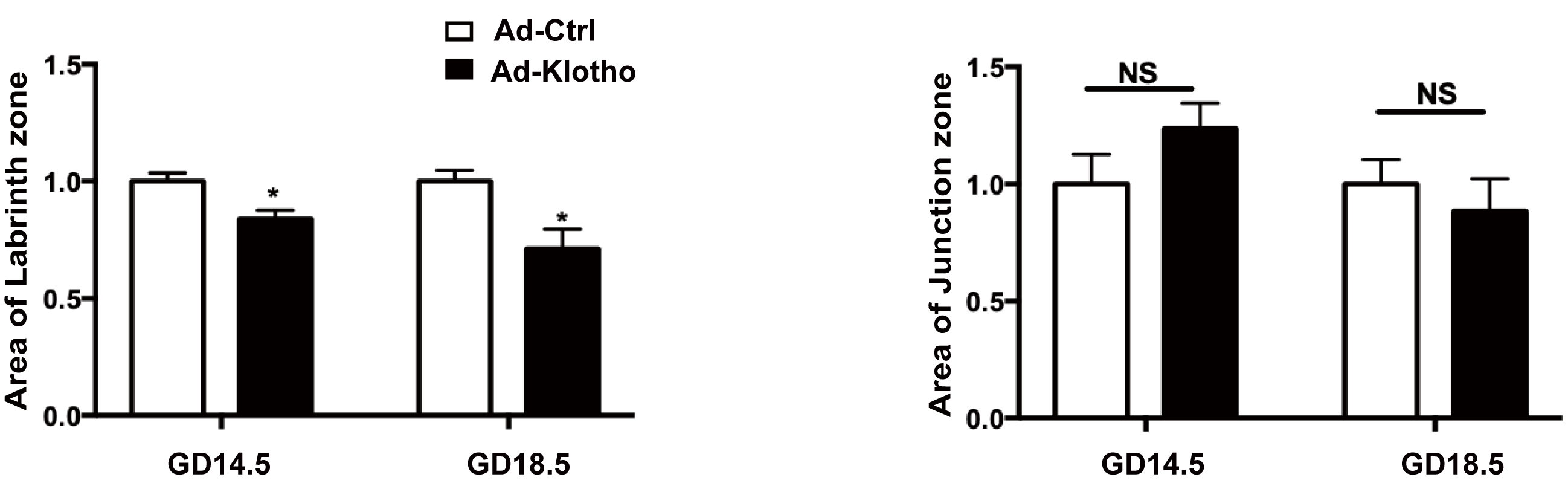


**Figure.S6** **The impact of α-Klotho deficiency on mouse placenta development.**

(a) Area of labyrinth zone (Lz) was analyzed ;(b) Area of junctional zone (Jz) was analyzed. GD14.5 (Ad-Ctrl, n=6; Ad-Klotho, n=6), GD18.5 (Ad-Ctrl, n=5; Ad-Klotho, n=5). NS, nonsignificant. All data are presented as the means ± SEM. *P, < 0.05, **P, <0.01, ***P. Mann-Whitney U test. All experiments are performed in triplicate.

**a b**


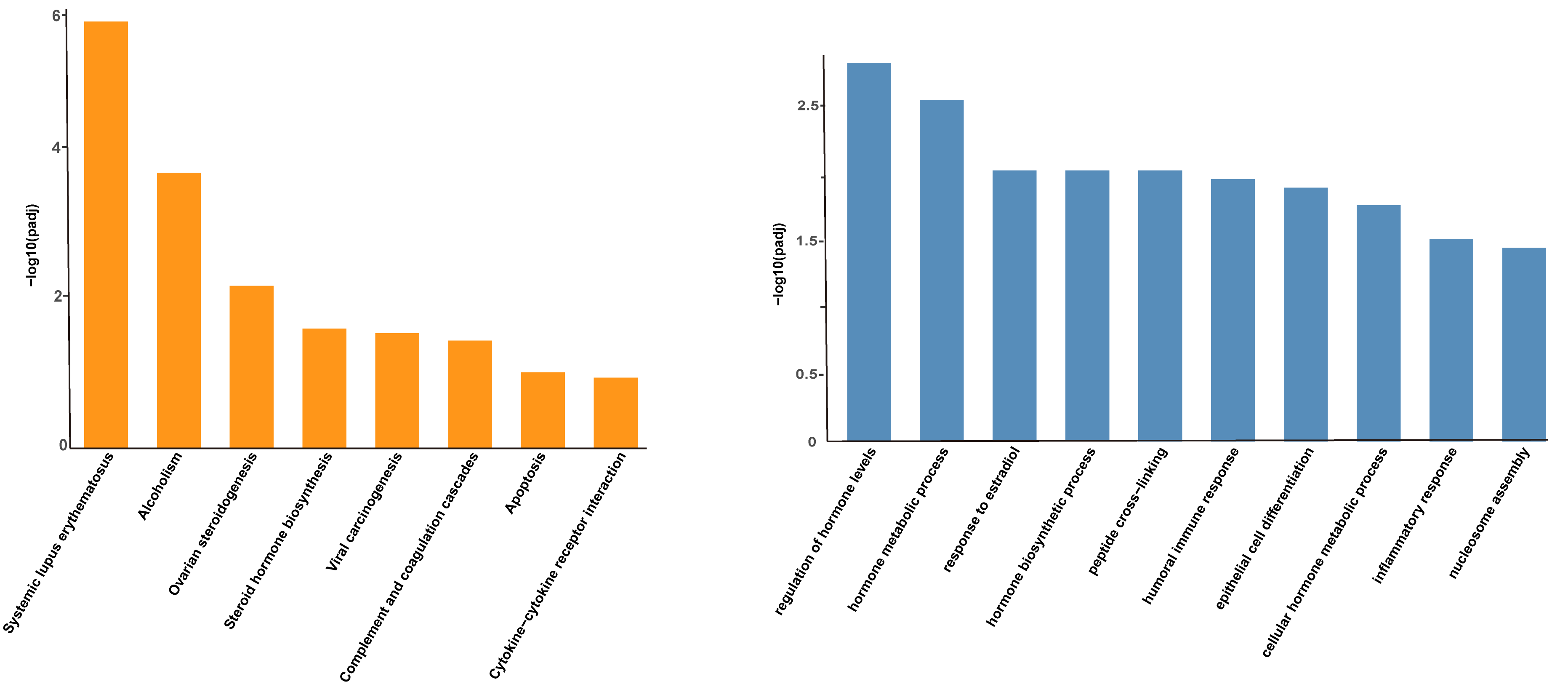


**Figure.S7** **GO and KEGG analysis for up-regulated genes in sh-KL JAR cells.**

(a) GO analysis; (b) KEGG analysis.


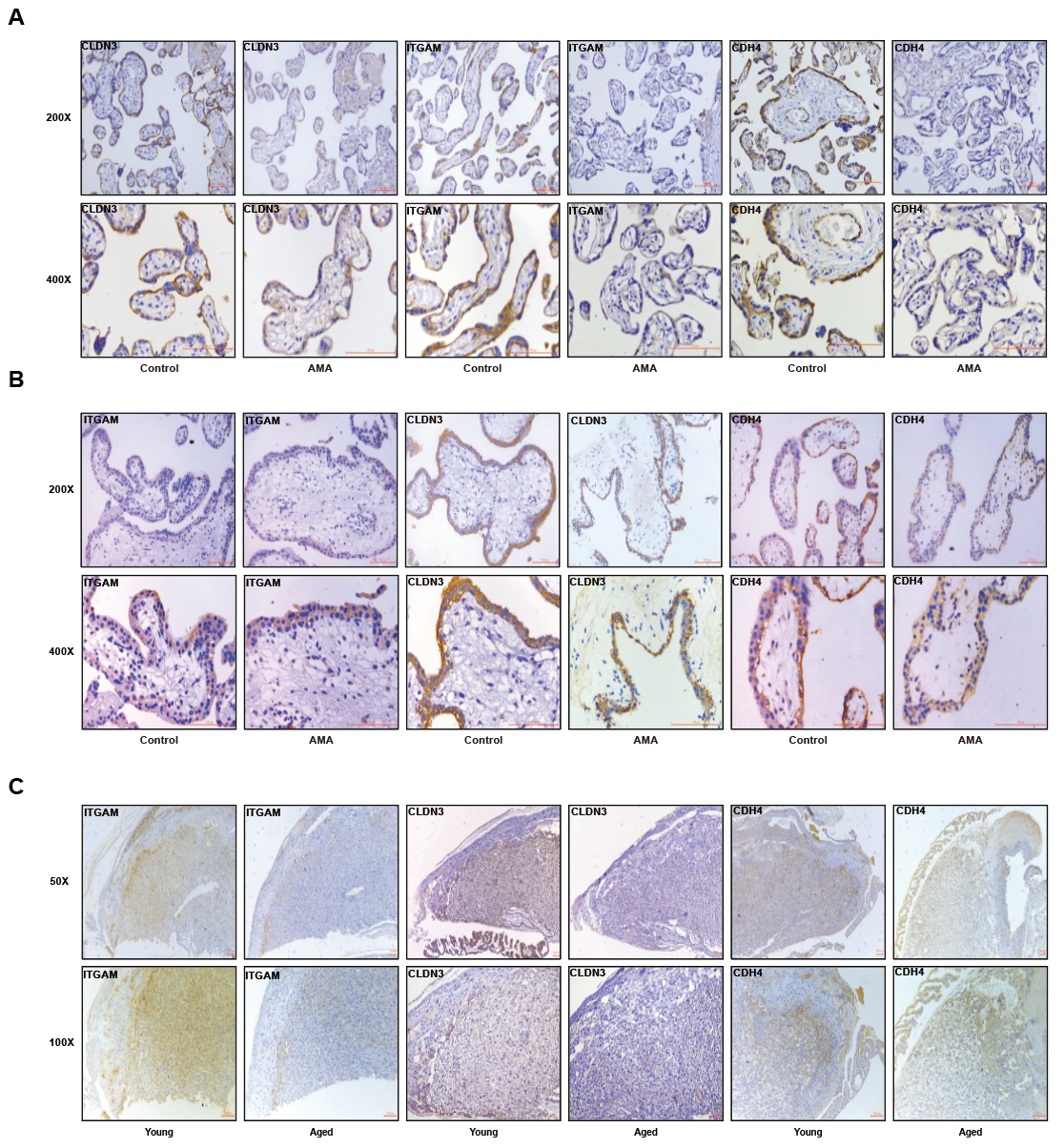


**Figure.S8** **CAMs expression in human and mouse placentas.**

(a) IHC staining of CDH4, CLDN3 and ITGAM in human term placentas collected from AMA and controls; 200X magnifications and 400X magnification of images were depicted. Scale bars, 100μm; (b) IHC staining of CDH4, CLDN3 and ITGAM in human villi collected from AMA and controls; 200X magnification and 400X magnification of images were depicted. Scale bars, 100um; (C) IHC staining of CDH4, CLDN3 and ITGAM in placentas collected from young and aged mice on GD18.5; 50X magnification and 100X magnification of images were depicted. Scale bars, 100μm.


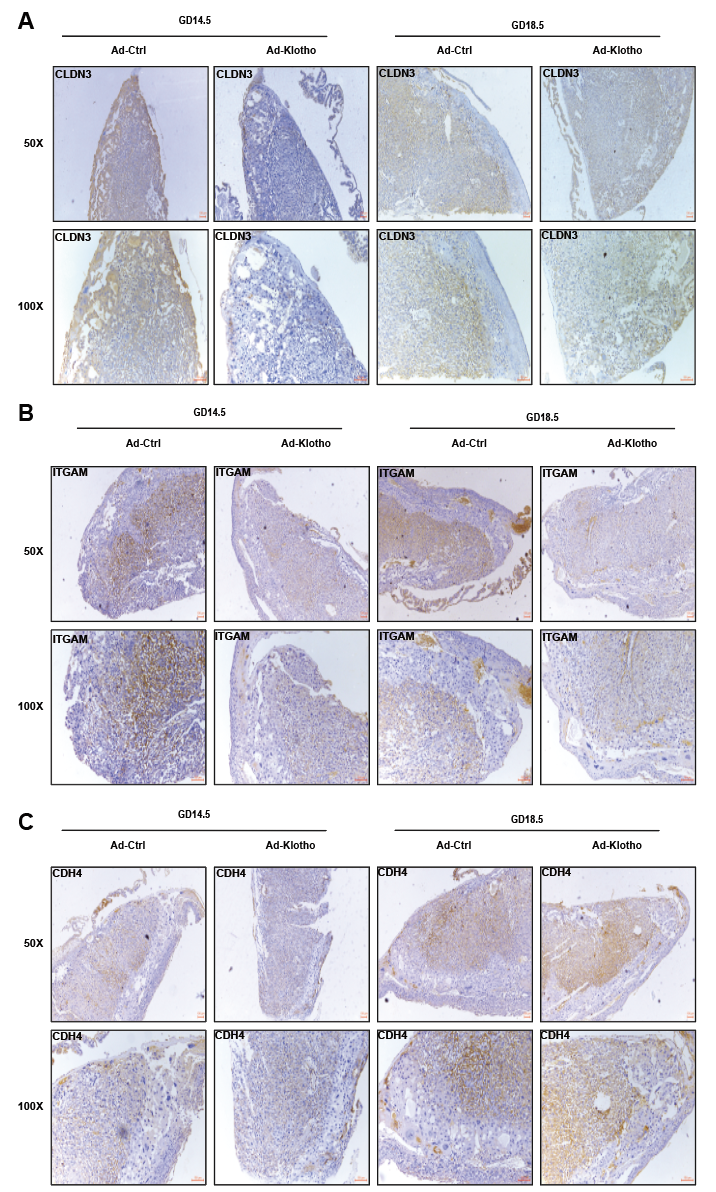


**Figure.S9** **CAMs expression in mouse placentas.**

IHC staining of (a) CLDN3; (b) ITGAM; (c) CDH4 in placentas collected from Ad-Ctrl or Ad-Klotho mice on GD14.5 and GD18.5. 50X magnification and 100X magnification of images were depicted. Scale bars, 100μm.


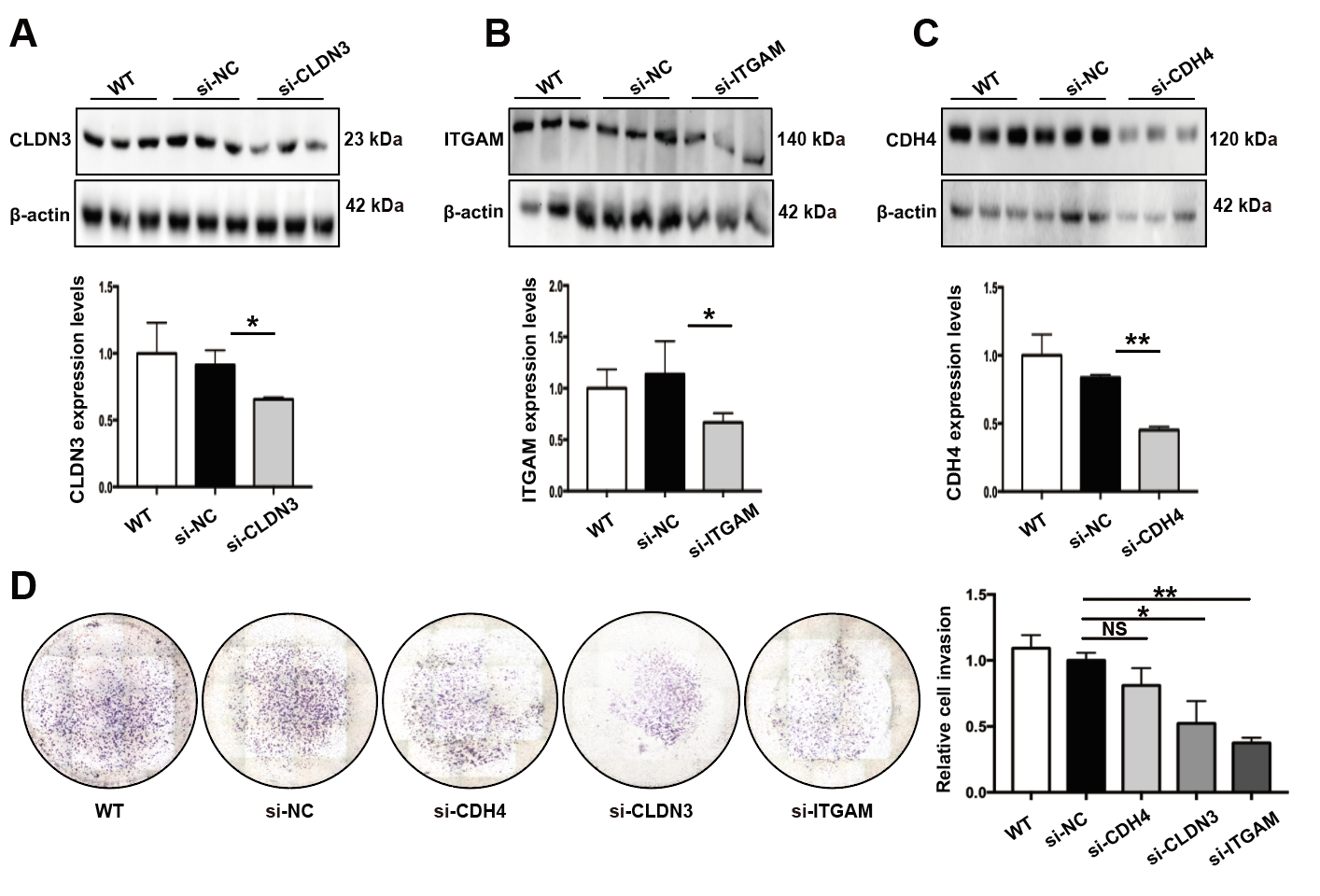


**Figure.S10 Loss of CAMs compromises invasiveness in JAR cells.**

(a) Western blotting of CLDN3 in JAR cells after 48h of si-CLDN3 transfection; (b) Western blotting of ITGAM in JAR cells after 48h of si-ITGAM transfection; (c) Western blotting of CDH4 in JAR cells after 48h of si-CDH4 transfection; (d) Transwell invasion assay in JAR cells in the presence of si-CDH4, si-CLDN3, si-ITGAM or si-NC. WT, wild-type; si-NC, negative control cells transfected with scramble siRNA; si-CDH4, cells transfected with siRNAs targeting CDH4; si-CLDN3, cells transfected with siRNAs targeting CLDN3; si- ITGAM, cells transfected with siRNAs targeting ITGAM. All data are presented as the means ± SEM. *P, < 0.05, **P, <0.01, ***P, <0.001. NS, nonsignificant. Mann-Whitney U test. All experiments were performed in triplicate.


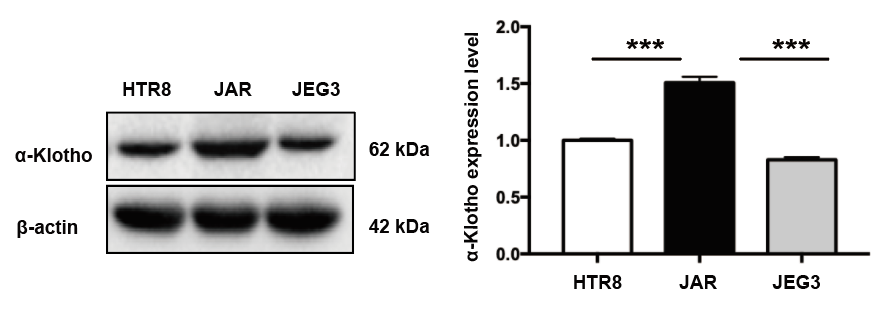


**Figure S11 Western blotting of α-Klotho in JAR, HTR-8/SVneo and JEG3 cells**

Western blotting of α-Klotho in JAR, HTR-8/SVneo and JEG3 cells. All data are presented as the means ± SEM. *P, < 0.05, **P, <0.01, ***P, <0.001. One-way ANOVA. All experiments were performed in triplicate.


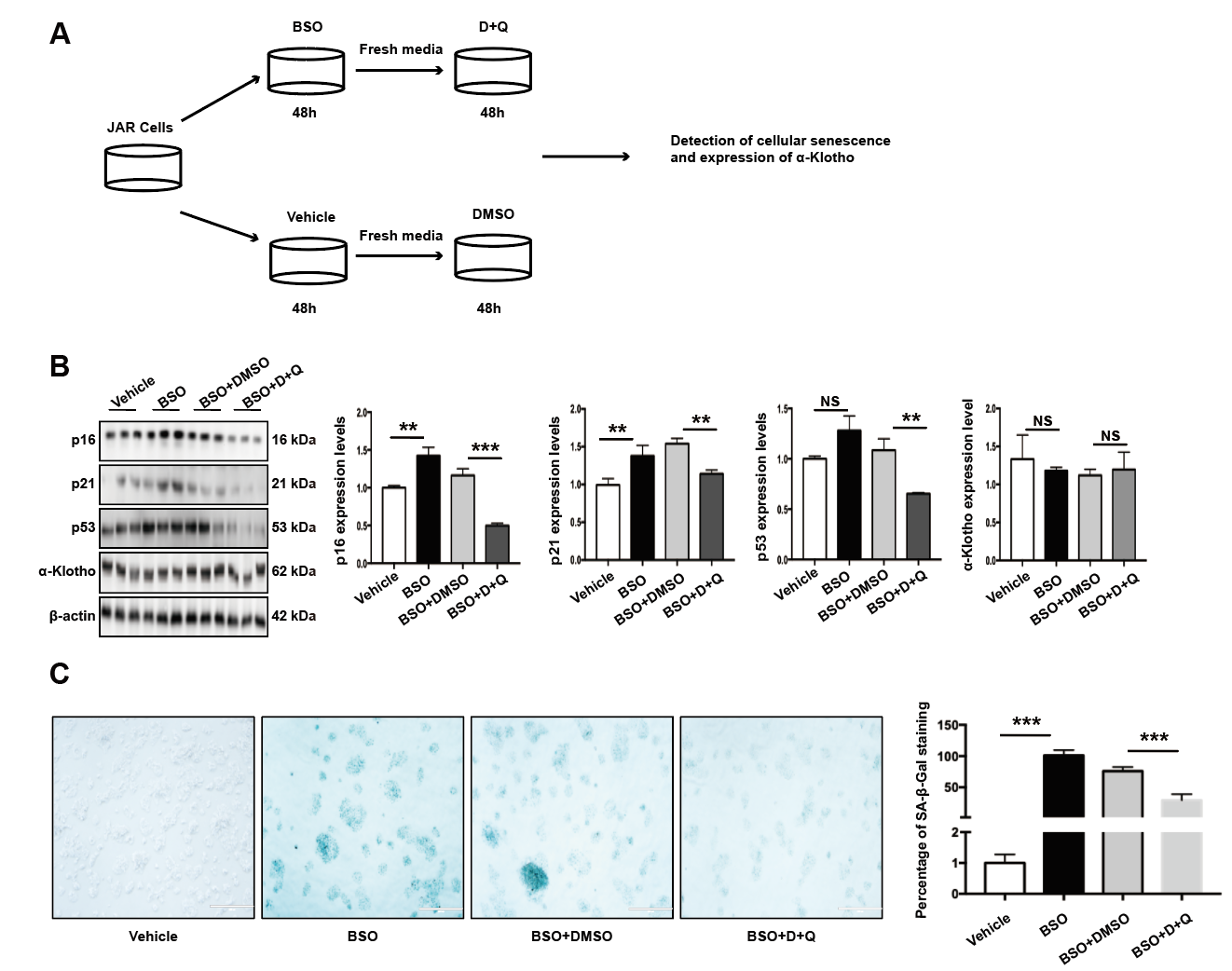


**Figure S12 The expression of α-Klotho was not affected by senescence.**

(a) Flow diagram of the experimental design; (b) Western blotting of α-Klotho, p21, p53 and p16 protein expression in JAR cells; (c) Representative SA-β-Gal staining in various JAR cells. Scale bars, 200μm; BSO, JAR cells treated with BSO for 48h; Vehicle, negative control JAR cells treated with water for 48h; D+Q, Dasatinib + Quercetin; DMSO, dimethyl sulfoxide; BSO+D+Q, senescent JAR cells (induced by BSO for 48h) treated with D+Q for 48h; BSO+DMSO, senescent JAR cells (induced by BSO for 48h) treated with DMSO for 48h; All data are presented as the means ± SEM. *P, <0.05, **P,<0.01, ***P,<0.001, one-way ANOVA. All experiments were performed in triplicate.


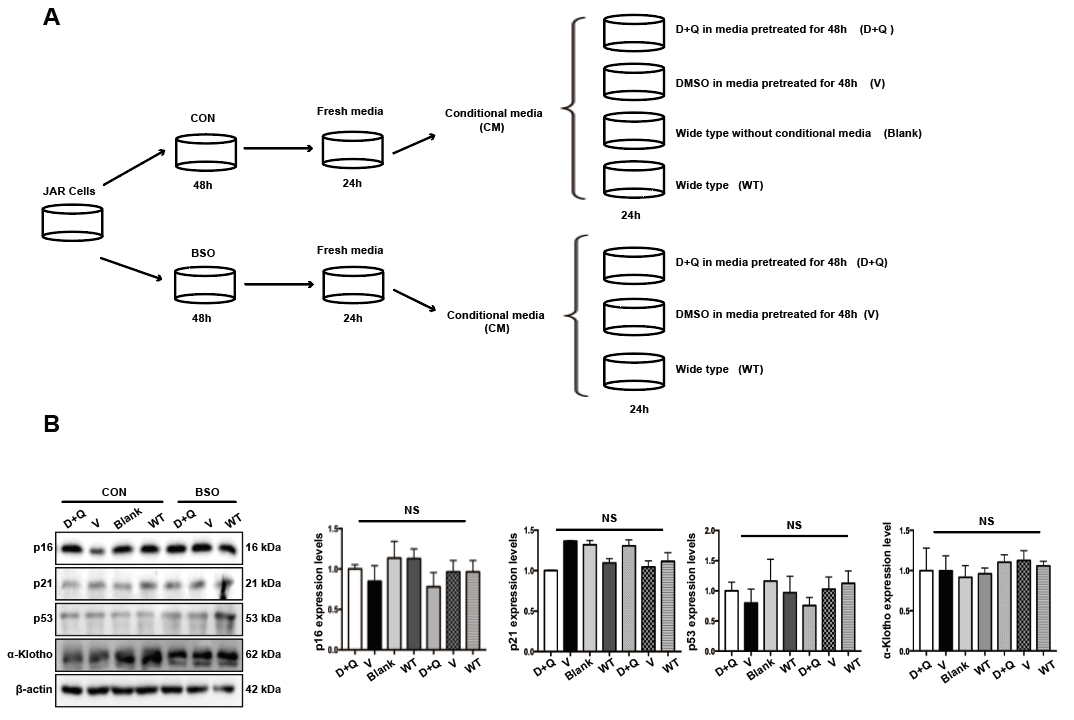


**Figure S13 Non-senescent JAR cells could not be affected by senescent JAR cells**

(a) Flow diagram of the experimental design; (b) Western blotting of α-Klotho, p21, p53 and p16 protein expression in JAR cells. Con, negative control JAR cells treated with water for 48h; BSO, JAR cells treated with BSO for 48h; D, Dasatinib; Q, Quercetin; WT, wide type; D+Q, D+Q in media pretreated for 48h; CM, conditional media; V, DMSO in media pretreated for 48h; Blank, wide type without conditional media; All data are presented as the means ± SEM. *P, <0.05, **P,<0.01, ***P,<0.001, one-way ANOVA. All experiments were performed in triplicate.


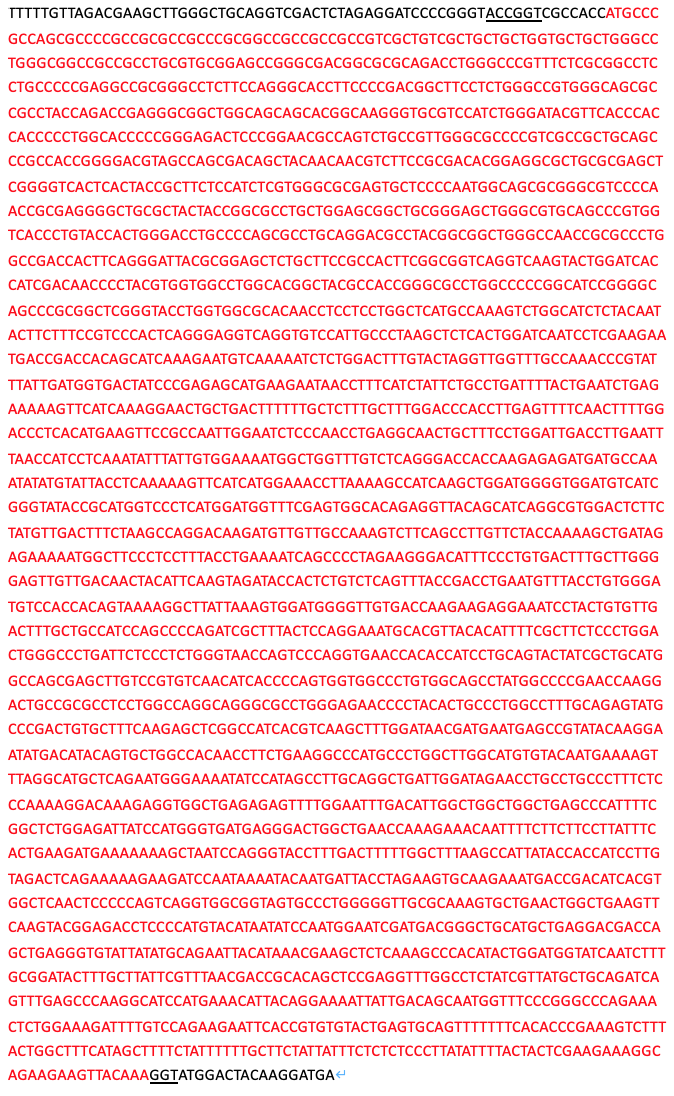


**Figure.S14 Detailed sequences of the α-Klotho-overexpression lentivirus.**

Black fonts, vector sequences; Underlined fonts, restriction enzyme cutting sites; Red fonts, insertion sequences.

**Supplementary methods**

**Induction and elimination of senescence in JAR cells**

Quercetin (Q) and D,L-Buthionine-(S,R)-sulfoximine (BSO) were purchased from

MedChemExpress (MCE, shanghai, china), and Dasatinib (D) were purchased from (sigma). We induced senescence in JAR cells by the use of 100mM BSO for 48h(Xiao et al., 2020). Moreover, JAR cells pretreated with BSO were then treated with D+Q (50nm + 10μm) for 48h (Xu et al., 2018; Zhu et al., 2015) to eliminate the senescent cells. Experimental design was depicted in Figure. S12.

Furthermore, 100mM of BSO was applied onto JAR cells for 48h to induce global senescence, and then the culture medium has been replaced. The cells were incubated with fresh culture medium for another 24 hours to collect the secretome. Then the conditioned culture medium (CM) of senescent JAR cells was applied onto non-senescent JAR cells (pretreated with D+Q) for 24 hours. After incubation, we detected cellular senescence and expression level of α-Klotho. Experimental design was depicted Figure. S13.

**Reference**

Xiao, Y. Z., Yang, M., Xiao, Y., Guo, Q., Huang, Y., Li, C. J., . . . Luo, X. H. (2020). Reducing Hypothalamic Stem Cell Senescence Protects against Aging-Associated Physiological Decline. *Cell Metab, 31*(3), 534-548.e535. doi:10.1016/j.cmet.2020.01.002

Xu, M., Pirtskhalava, T., Farr, J. N., Weigand, B. M., Palmer, A. K., Weivoda, M. M., . . . Kirkland, J. L. (2018). Senolytics improve physical function and increase lifespan in old age. *Nat Med, 24*(8), 1246-1256. doi:10.1038/s41591-018-0092-9

Zhu, Y., Tchkonia, T., Pirtskhalava, T., Gower, A. C., Ding, H., Giorgadze, N., . . . Kirkland, J. L. (2015). The Achilles' heel of senescent cells: from transcriptome to senolytic drugs. *Aging Cell, 14*(4), 644-658. doi:10.1111/acel.12344
